# Part I. Periscope of Hospital Reports, *English and Foreign*, with Commentaries

**Published:** 1827-01

**Authors:** 


### PRACTICAL MEDICINE;

BEING

### The Spirit of the Medical Journals,

*Foreign and Domestic;*

WITH COMMENTARIES.

### PART I.

### PERISCOPE OF HOSPITAL REPORTS.

"Neglecta reducit, sparsa colligit, utilia selegit, necessaria ostendit,—sic utile."—Baglivi.

### 1. M. ANDRAL ON CHRONIC GASTRITIS.

Memoire sur les Caracteres Anatomiques de la Gastrite Chronique. Par M. Andral, Fils.*

A Memoir on the Anatomical Characters of Chronic Gastritis. By M. Andral, Jun. [La Charité.]

Part I.—*Alterations of the Mucous Membrane.*

In the great majority of cases of chronic gastritis, dissection shews the mucous membrane of the stomach altered in various degrees from its natural appearance. Sometimes, however, no appreciable lesion can be discovered after death, the surface being of its natural colour, thickness, and firmness throughout. In such cases various diseased conditions of the subjacent cellular tissue are found—especially in that condensed and whitish membrane between the villous and muscular tunics of the organ. M. Andral concludes, from analogy, that, in such cases, the inflammation has extended from the mucous membrane to the subjacent tissue, leaving its former seat in apparent integrity. He illustrates this supposition by the following analogical facts. An individual is attacked with enteritis or colitis. If he dies in the acute stage, the mucous membrane alone will be found affected, either by redness, softness, ulceration, or other alteration. If he survives for a certain period, until after the inflammation has become chronic, the intestine presents three different states: 1mo. The inflammation may be confined to the mucous membrane only; 2ndo. The mucous membrane and the subjacent tissues may be affected in equal, or in very disproportionate degrees, presenting softness of

* Repertoire Generale, &c. No. 1 and No. 2, 1826.

structure, alterations of colour, in points, stripes, or patches—or even in ulcerations, in various stages of development or cicatrization, the same as we see in the mucous membrane lining the mouth or pharynx. In some of these instances, the mucous membrane would seem to be in a mending condition, while the sanative process had not yet commenced or advanced in the subjacent tissue. 3tio. The mucous membrane returned to a perfectly sound state, while traces of disease are still cognizable in the cellular membrane underneath.

In the mucous membrane of the lungs, the same succession of phenomena may be seen: thus, in acute bronchitis, the inflammation is bounded to the mucous membrane; in chronic bronchitis, the lining membrane and the tissue underneath are both affected, in various degrees; and, finally, at a more remote period, we shall find the mucous membrane become sound in structure, at least in appearance, while diseased conditions still obtain in the neighbouring parts. If these analogies are not quite satisfactory, M. Andral resorts to a more tangible example: thus, after an inflammation, more or less intense, of the conjunctiva, and after this membrane has begun to resume its accustomed transparency, the cellular tissue connecting it with the sclerotic will sometimes continue inflamed, infiltrated with pus, thickened, or the seat of various incipient alterations of structure. The same may be observed of old gonorrhœas, where the mucous membrane of the urethra will have regained its pristine state of integrity, the subjacent cellular tissue remaining thickened, indurated, and causing strictures of the passage. Other examples, of the same tendency, may be cited from inflammations of the serous and synovial membranes, where these last are become natural in appearance, the traces of disease still subsisting in the contiguous structures. Applying these analogies, then, to the stomach, we may conclude that the inflammation (the traces of which can only be found in parts beneath the mucous membrane) had its primary seat in this membrane itself. M. Andral avers that he has had opportunities of proving, by ocular demonstration, these transitions and changes, in cases that happened to be cut off in various stages of disease.

The next question is this:—When the mucous membrane of the stomach is returned, in appearance, to a state of integrity, the traces of disease still existing in the parts beneath, is the *appearance* a proof of *real* integrity in the membrane? It is to be observed that, notwithstanding this apparent sane state of the membrane, in the eye of the anatomist, the physician will find that the *function* of the part is not restored to health. The digestion will be slow and painful, and the surface of the stomach incapable of performing those changes in the food which constitute perfect chymification. This observation applies, also, to other parts, besides the stomach. Thus, in some individuals, who had presented all the symptoms of chronic bronchitis, with copious puriform expectoration, our author has found the mucous membrane of the larynx, trachea, and bronchia, in apparent integrity, and without the smallest vestige of even redness, so that its pathological condition could only be inferred from the disordered function during life.

But a sound condition of the mucous membrane of the stomach in chronic gastritis is a rare occurrence. Most frequently it presents divers alterations in consistence, thickness, and form. The alterations in colour are very often the same as in acute gastritis. These are redness, grey-slate colour, (grise-ardoisée) brownness, and blackness, in various degrees of intensity. We need not enter into M. Andral's speculations on the cause of these different colours; but if any one should doubt that they *are* signs of previous chronic inflammation, he submits the following facts for their consideration.

1mo. If the symptoms during life have been well observed in such cases, they will always be found to have been those of chronic gastritis —at least such is the result of his observations in La Charité. But these symptoms often escape observation, if great attention be not paid, because they are not, in fact, very prominent; and because chronic gastritis is often complicated with other maladies which absorb the attention of the practitioner.

2ndo. But in the greater number of these cases of discoloration in the mucous membrane, other alterations will be observed which cannot but be considered as proofs of previous gastritis, such as thickening, induration, vegetations, &c. while the subjacent tissues present unequivocal traces of phlogosis.

3tio. In certain cases of ulceration of the stomach, and still more frequently in ulceration of the intestinal canal, we find the borders of the ulcers discoloured in the manner above-mentioned.

4to. We must not attribute the grey-slate discolorations to putrefaction, *post mortem*, since, in many bodies that are highly putrid, we find marks of putrefaction, but never the grey-slate or brown color of chronic gastritis. Some have attributed this peculiar appearance to the operation of the gazeous contents of the stomach or bowels—especially to the extrication of sulphuretted hydrogen gas—but this gas has never been demonstrated in the stomach where the above appearance is most common. We shall not follow our author through all his minute descriptions of these discolorations; but it may be observed that the brown and slate-coloured appearances are very rare in acute gastritis, and the most common of all in chronic. Hence M. Andral thinks that a mere inspection of the color alone may generally determine which of these inflammations existed previously to death. But it is to be remembered that, although the grey-slate colour is rare in acute gastritis, it is by no means uncommon to find the mucous membrane of a bright red colour in chronic inflammation of the organ.

In some cases of unequivocal chronic gastritis, M. Andral has found nothing but some large stripes of thickened and condensed mucous membrane, the colour being actually paler in those parts than in the rest of the stomach. In one case particularly, which recently occurred in La Charité, the local and general symptoms of chronic gastritis were so marked, that a cancerous affection was suspected, the patient having often vomited black matters like coffee grounds—and yet, on dissection, the internal surface of the stomach presented only one patch of a milky white colour, with some thickening and induration of the membrane. In other instances, this milky whiteness was found to coincide with a swelling and softening of the mucous membrane, having vessels traversing it, and also some red spots scattered over its surface.

In almost every case where the stomach has been the seat of long-continued inflammation, dissection shews some alteration in the consistence of the mucous membrane, consisting of either induration or softening. The *induration* is one of the best marks by which chronic may be distinguished from acute gastritis, whether as regards the membrane itself or the contiguous tissues. Softening of the membrane is a consequence equally of chronic and acute gastritis—but *induration* appertains exclusively to the *former*. This induration may be either general or partial. It may co-exist with a natural colour of the membrane—with a white colour—or with a grey or brown colour—but never with a red or vermillion colour. Often as this *induration* is found in the stomach, it is less frequent than the opposite condition—softening. This last is the most frequent of all organic changes found in the stomachs of those who have sunk under chronic diseases of various kinds in the hospitals. Hence, if it can be proved (as it presently will) that this softening of the mucous membrane is the product of inflammation, it follows that gastritis, acute or chronic, is a very common disease—much more common than has hitherto been supposed, whether it exists as a primary affection, or only as a complication with, or supervention on other maladies.

This softening has been so well described by M. Louis, that our author does not deem it necessary to go into its description here:—he observes, however, that it may be divided into three principal grades, viz. in the first degree, the membrane, though much softened and easily reduced to a pulp between the fingers, yet preserves some appearance of consistence before it is scraped with the scalpel:—in the second grade, we find, in place of mucous membrane, only a pulp, of a white, grey, or reddish colour, which one would easily mistake for a layer of mucus spread over the cellular tunic. In the third degree, this semi-liquid pulp has disappeared, and the subjacent cellular tissue is left naked, in spaces of greater or lesser extent. One of the most remarkable examples of this was seen in the case of a man, aged 36 years, who died phthisical in La Charité in June, 1824. During the three months which he spent in the hospital, he had no vomiting, but complained of complete loss of appetite, an habitual sense of tightness in the epigastrium, changing into actual pain on taking food, and sometimes even on taking simple drink. Wine produced nausea, and a sensation of burning in the region of the cardiac orifice of the stomach, thence spreading up along the œsophagus to the pharynx. This man died, and on dissection, there was only to be found the *debris*, as it were, of the mucous membrane. From the cardia to the pylorus, the sub-mucous cellular tissue was quite naked, being stripped of its mucous membrane, but still preserving its accustomed white colour, being merely a little thickened. In some spots, there were the remains of the villous coat, recognized by a pale red colour, and some degree of prominency. There were also tubercles in the lungs, and ulcerations in the intestines.

M. Andral confirms the observations of M. Louis,* respecting the state of the mucous membrane of the stomach in those who die of phthisis; but it is curious that in these cases, the *softening* of the membrane is confined to certain portions of the stomach, especially the cul de sac, and the splenic extremity of that organ—while the *induration* is more commonly found about the pyloric orifice.

Here M. Andral starts the question—" are these softenings of the mucous membrane to be considered in all cases as the product of inflammation?" Some writers have left the subject undecided—some have attributed the phenomenon to a peculiar morbid process different from inflammation—while others feel quite confident that a mucous membrane softened is a mucous membrane inflamed. The question is of importance, and M. Andral dedicates a few pages to the discussion.

What do we learn from the anatomical characters? They teach us that, in the greater number of cases where there is softening of the mucous membrane, there will be also found other unequivocal indications of phlogosis, as redness, &c. It is also to be remarked, that if we examine the effects or consequences of inflammation in other structures of the body, we shall find *softening* a very common occurrence. The inflamed cellular membrane is softened as well as reddened and infiltrated with pus. M. Dupuytren has long ago pointed out the extreme lacerability of the cellular sheath of arteries, when these vessels have been in a state of inflammation, whence proceeds the premature division of this sheath by the ligature. The serous membranes when inflamed, become softened. So does the cutaneous tissue. If we examine around a variolous pustule, we shall often find the parts so softtened, that they are lacerated by the least force. At the termination of synovial inflammations, acute or chronic, who has not observed the ligaments and other parts surrounding the articulation, reduced almost to a pulp? Even the cartilages, in these cases, are sometimes rendered a pultaceous mass. The periosteum at first is hardened, but ultimately softened by inflammation. In what cases do we find softening, and finally ulceration in the transparent cornea? It is almost always consecutive of inflammation of the conjunctiva. In the parenchymatous structures, one of the first effects of inflammation is to diminish the force of cohesion. Lallemand and many others have demonstrated the softenings of the brain to be consequences of inflammation. The lungs and the liver often present softenings, as well as indurations, the products of inflammation.

But in some cases of softening of the gastric mucous membrane, the pale colour is preserved, so that the part, on a superficial inspection, appears perfectly sound. Are these conditions also to be considered as the effects of preceding inflammation? M. Andral hesitates not to

* See volume two of this Series, p. 173-181. Also Vol. 3, p. 530, and Vol. 4, p. 492-497.

answer in the affirmative. It is to be remembered, that redness is not an infallible accompaniment of inflammation. The serous membranes will be so inflamed as to throw out pus, and yet preserve their pale colour. They will not *redden* in inflammation, but they will *soften.* No one will deny that the induration of cellular membrane around old ulcers is a product of inflammation—yet this induration often presents a perfect whiteness—" une parfaite blancheur." The softening of the transparent cornea is often unpreceded and unaccompanied by redness. Paleness of these softenings of the gastric mucous membrane then, is no proof that the phenomenon is not a sequence of inflammation. So much for morbid anatomy:—let us now look to symptomatology.

There is a certain proportion of instances in which the symptoms of inflammation are observed during life, especially where the gastritis has been acute, and where the mucous membrane has been found red as well as softened. When, on the other hand, the affection is chronic, two states of the case may present themselves:—1mo, there may be symptoms of inflammation, more or less marked, such as epigastric pain, augmented by the ingestion of aliment or drink—nausea or vomiting, &c. These are symptoms which we observe during life, in cases where dissection has shewn unequivocal evidence of inflammation in the stomach, as thickening, ulceration, &c. of the inner membrane. 2ndo. we may find the mucous membrane considerably softened, on dissection, while during life, there were few, if any symptoms of gastritis. There may have been no vomiting—no loss of appetite—no pain—no thirst—no disturbance of the circulation. The patient merely complains that the digestion is more or less uneasy and imperfect, and that he loses flesh and strength. But then let it be observed, that it is not in cases of softening of the stomach alone that we find this absence of symptoms characteristic of inflammation. The stomach has often been known to be the seat of extensive and deep ulcerations, large fungous tumours, and various other degenerations of structure, without any remarkable disturbance of its functions, more than what are mentioned above, as attendant on softening of the mucous membrane. It is therefore necessary to conclude either that these softenings are sometimes the effect of inflammation and sometimes not—which would be absurd—or to conclude that they are the products of inflammation at all times, though not always accompanied by evident symptoms of such state in the living body. This last proposition is the only one that is admissible:—and this admitted, we are forced to conclude that, according to the laws of pathology, every phlegmasia may exist under two forms—one, *manifest*, and one more or less completely *latent* or concealed. This is a law which morbid anatomy alone could ever have revealed—for if there never had been dissections, we never should have suspected internal inflammation where the external symptoms were absent. "Thus the study of symptoms as well as of morbid anatomy leads us to conclude that softening of the gastric mucous membrane is the product of inflammation."

Looking to etiology, we shall find that the greater number of agents in the production of these softenings, belong to the class of irritants. Poisons introduced into the stomach of animals produce the phenomena in question. Our author found this softening, in a very high degree, in the stomach of a child to whom sulphuret of potash had been given three months previously, for the cure of croup. From the time this medicine had been given, the child had vomitings, and daily emaciated. At La Charité, our author has had frequent opportunities of opening the bodies of those who had been addicted to intemperance in alcoholic liquors, and one of the most common changes found in their stomach, is this softening, with redness or whiteness of the mucous membrane. That softening, which M. Cruveilheir has denominated "*gelatiniform*," observed in the stomachs of children that have been prematurely weaned, and then fed on gross aliments, may be readily placed in the same class with that of which we are treating. In other organs, we constantly see these softenings produced under the influence of unequivocal causes of irritation. Thus, after blows or falls on the cranium, the brain inflames, and ultimately becomes softened. So when foreign bodies are introduced and remain in parenchymatous structures; or when morbid growths are there developed, the parenchyma becomes irritated by their presence—inflames—and finally softens. This we frequently see in the brains of children around tubercles there. Undoubtedly these softenings sometimes become manifested in the stomach and other organs, without any apparent preceding cause of irritation; but if they exhibit the same anatomical characters and the same symptoms as those developed after evident causes of irritation have been applied, we may safely conclude that they are, in both instances, of the same nature. Could we maintain the existence of an inflammatory and a non-inflammatory arachnitis, because the one was produced under the influence of unequivocal causes of irritation, and the other without any apparent cause at all? If it be objected that these softenings of the stomach and brain are seen in people of different ages, the old and the young—the feeble and the robust; the same may be said of inflammation itself, which happens in the weak as well as in the strong—with this difference, that the local inflammation is announced by a different train of symptoms in the two classes of subjects. Thus in a young, plethoric, and irritable person, endued with great nervous susceptibility, a very small softening of the stomach will produce a great general re-action in the system, followed by intense fever, delirium, convulsions, disturbance of all the functions, and sudden death. In individuals placed under opposite conditions, this softening may commence, run its course, and disappear, without exciting any other symptoms than those attending what is called indigestion. The former may destroy life in a few days—the latter may last for years in a chronic state. It is thus that softening of the brain, in a young person, will be attended with convulsive movements in the system, while in old subjects it will only produce simple paralysis.

Let us now look to therapeutics. We shall find that tonics and stimulants applied to a softened mucous membrane always aggravate the symptoms, and often change into evident gastritis what was before only an obscure dyspeptic affection. On the contrary, antiphlogistic remedies mitigate the symptoms.

In respect to the volume or thickness of the mucous membrane of the stomach, it may be of its natural or normal dimensions—it may be more or less augmented in thickness—or it may be attenuated. In the first condition, which is not very rare, we find the membrane either of a red or brown colour, or of various colours, and more or less softened in texture. The augmentation of thickness in the mucous membrane is of very frequent occurrence in chronic gastritis. It is sometimes accompanied by a softening, sometimes by an induration of the membrane. In the *first* case, the thickening ought rather to be called a tumefaction of the parts, occasioned by an afflux of fluids under the influence of an inflammatory stimulation, in the same way as the reticular tissue of the skin is swelled by the irritation of a blister. This phenomenon (tumefaction with softening) is more frequently the consequence of acute than of chronic gastritis, though occasionally seen in the latter disease. On the other hand, *augmentation of volume with induration*, is exclusively confined to chronic inflammation, and furnishes one of the least equivocal evidences of the previous existence of that malady. In this case, there is a real increase of density—a veritable *hypertrophia* of the mucous membrane, the consequence of morbid or excessive nutrition, when this tissue is long the seat of irritation and inflammation, and consequently receiving more blood than in a state of health. But if, instead of this simple hypertrophy of the membrane, a softening or ulceration takes place, the law of excessive irritation will be changed, or absolutely perverted.

Whether thickening of the mucous membrane be accompanied by softening or induration, it may invade at one time, a great extent of surface, or be confined to a few insulated points. These points may be scarcely perceptible to the eye, and only appreciable when the membrane is detached. From this species of disorganization result those appearances which have been termed exanthemata, vegetations, and tumours of various forms, textures, and dimensions. M. Andral thinks that great error has been committed by giving to certain of these forms appellations which convey the idea of a specific nature, and draw the attention of the physician from the original cause that produced them—irritation and inflammation, chronic or acute, of the mucous membrane.

In respect to the intimate texture of these morbid growths, they may be divided into two classes. In the first may be ranged all those tumours, vegetations, &c. which appear to be of the same texture as the indurated or softened membrane itself. In the second class are to be ranged those tumours, whose structures do not correspond with sound or unsound membrane. The first class, he thinks, will be readily attributed to an inflammatory process. The nature of the process, however, by which the morbid growths comprehended in the second class, for instance, encephaloid tumours, are built up, admits of some doubt; and although M. Andral is inclined to place chronic inflammation as the cause of both classes, he admits that more facts are wanting before positive proofs can be adduced of the truth of his doctrine.

Finally, there are cases where the mucous membrane of the stomach, instead of being thickened, is actually attenuated, and has undergone a real atrophy. It is especially about the great *cul de sac* of the stomach that this appearance, as well as the morbid softening, is usually seen. In two cases only has our author found this attenuation in the neighbourhood of the pylorus. Can this atrophy or attenuation, says he, like hypertrophy and softening, be attributed to inflammation? He concludes in the affirmative. A woman, aged 36 years, died in the Charité, in the month of March, 1825. During the last three months of her existence, she had frequent vomitings. On dissection, the stomach presented several red points and patches, and, in the splenic extremity, there were softenings and attenuations, situated both in the red patches and in the intervening spaces. Still our author admits that this attenuation of the mucous membrane may take place in the same way as atrophy of the muscles in phthisis, without any actual inflammation.

Ulceration of the mucous membrane of the stomach is infinitely more rare than that of the small and large intestines. Ulceration is an extremely rare phenomenon in acute *gastritis*, unless where poisons have been introduced; while, in acute *enteritis*, ulceration is very common. On the other hand, chronic gastritis pretty often shews ulceration of the lining membrane, generally in one spot only, of greater or less diameter, around which the mucous membrane may be sound or unsound.

### Part II.—*Alterations of the other Tissues or Coats of the Stomach.*

The follicles, situated under the mucous membrane of the stomach, become the seat of important morbid alterations, according to our author. These lesions respect the volume, the structure of the parietes, and the secretions of the follicles. Stomachs are sometimes found where the most attentive examination of their internal surface can scarcely discover any crypts—which absence of follicles will equally obtain in stomachs apparently sound, and in those where acute or chronic inflammation had evidently existed before death. On the inner surface of other stomachs, the follicles are much more visible. In a state of real hypertrophia, they present themselves like rounded granulations, insulated or agglomerated, in various parts of the stomach. It is not probable that, in such cases, those follicles are entirely new formations; but, too small in their natural state to be appreciable by the naked sight, they become so by inflammation. These preternatural developments of the follicles are either partial or general. In the former case, they are principally observed about the cardiac orifice, or near the pylorus. In this last situation, the crypts are sometimes so much enlarged and agglomerated, that the internal surface of the stomach resembles the structure of the duodenum, especially the first flexure of this organ.

When the development of the crypts is general, two varieties are presented on the internal surface of the stomach. Sometimes a great number of granulations are extended over the whole surface, of various sizes, white, grey, red, or brown, in the centres of which it is often possible to perceive a small orifice surrounded by a small red or black circle. Sometimes these crypts are so greatly magnified, that they have a nipple-like appearance, which would induce one, at first view, to suppose there was a morbid structure of the mucous membrane itself; which may sometimes be the case; but, on attentive examination, it will generally be found that the phenomenon is owing to enlargement of the follicles. This mammillary appearance is usually accompanied by a grey or brown colour of the mucous membrane, and, during life, these are the symptoms of chronic gastritis. More than once our author has found only this mammillary structure, when there had been every reason to believe that cancer of the stomach existed, from the symptoms, such as lancinating pain in the epigastrium, yellowish tint of countenance, marasmus, vomiting of food, and also of black matters, &c.

In the horse's stomach certain appearances sometimes present themselves, which M. Andral thinks deserving of notice. They are roundish tumours on the mucous membrane, from the size of a cherry or a walnut, to that of a large orange. The mucous membrane passes over these tumours, and in the centre of each is an orifice capable of admitting a probe, which passes readily into the interior. This interior is found not to be of solid structure, but a pouch filled with a liquid of varying kind and consistence—sometimes resembling mucus—sometimes pus—and sometimes of a grumous character, or resembling honey, or that which is squeezed from melicerous tumours of the skin. The parietes of these tumours are as variable as their contents. In some tumours they are very thin, and appear to be merely prolongations of the mucous membrane, covered outwardly by a layer of dense cellular tissue. In other tumours, this layer assumes a fibrous, and even a cartilaginous appearance. In these tumours, M. Andral has often found a great number of the ENTOZOÆ, having all the characters of the *nématodes* of Rúdolphi, being of a beautiful white colour, one or two lines in length, the thickness of a hair, and possessing great agility in their motions. These animals have been observed issuing from the central aperture, and spreading themselves over the interior of the stomach. They were not the products of any putrefactive process, since they were observed in horses opened the very instant after they were slaughtered. The constancy and regularity of the orifices in the centres of these tumours leave little doubt that they are the mouths of dilated follicles. This would be presumed from the examination of the larger ones—but, when they are traced through their various gradations, all doubt vanishes, as we come at last to the follicles themselves in scarcely a state of enlargement. From these and other facts, our author is led to conclude that many of those tumours and other morbid growths which are considered as adventitious or heterogeneous structures, are only excessive or morbid developments of natural tissues. In his Clinique Medicale (vol. 3) M. Andral has endeavoured to shew that this is the case in pulmonary granulations, or incipient tubercles; and at the close of this memoir, the same will be attempted in respect to the principles of formation in certain diseases of the stomach, especially scirrhus.

The fluids secreted by a healthy stomach may be very much altered from their natural condition during chronic gastritis. An individual entered the CHARITÉ with all the symptoms of this disease, having daily vomitings of some pints of a glairy white mucus, resembling the white of egg. There was this remarkable circumstance, that the patient never threw up any of the food or drink which was taken into the stomach. On dissection, nothing was found but a thickening (hypertrophie générale) of the mucous membrane, with a brownish tint in colour, and a strong development of the follicles.

Among the matters found in the stomach after death, (in chronic inflammation,) and of which prodigious quantities are sometimes thrown up during life, we may remark on the black or coffee-ground looking fluid, which has so often engaged the attention of pathologists. What is the nature of this fluid? Is it connected with any, and what special lesion of the stomach itself? Lately our author sent a quantity of this black matter, thrown up by a female in the quantity of a pint daily, to his friend, M. Lassaigne. It was found to contain much water, albumen, a free acid, of an organic nature, and to hold in suspension a colouring matter, insoluble in water, soluble in sulphuric acid, and presenting, when thus dissolved, a beautiful blood-red appearance. Submitted to calcination, it burned without swelling, and left a slight residue of a brick-colour, composed of oxide of iron, and traces of phosphate of lime, the same as is furnished by the colouring matter of the blood. Hence M. A. concludes, that the black matters ejected from the stomach in certain diseases, are owing to the presence of an organic element, having a great analogy to the colouring matter of the blood. Such is the conclusion come to also by M. Breschet, in his work on Melanosis. In the 18th volume of the Dictionnaire de Medecine (Art. *Melanose*) M. Andral has adduced fresh proof in support of M. Breschet's opinion. In a stomach which he recently examined, this colouring matter was found under two forms—one, in which it existed free in the stomach; the other, in which it was combined, in several places, with the mucous membrane, giving that tissue a beautiful jet black colour. The black matter, then, which is vomited in cancerous affections (for example) of the stomach, is to be considered, M. Andral thinks, as identical with the matter of melanosis, and like that to be constituted of a colouring principle analogous to the colouring matter of the blood.

It is highly probable, however, that this colouring matter is the product of a *peculiar secretion*, like some of the numerous colouring matters, blue, green, yellow, &c. which so richly tint the skins and coverings of various animals.

It is supposed that black vomiting is a characteristic sign of a cancerous state of the stomach, and that ejected black matters come from the ulcer. This is clearly a mistake; for similar vomitings have been met with where no such ulcer existed. Our author has observed this phenomenon in people whose stomachs presented, on dissection, tumours of the sub-mucous cellular texture, (the scirrhus of systematic writers,) without any breach of structure. In other instances, where black vomitings had obtained, during life, he has found merely a thickening of the mucous membrane, or a mammillary configuration of its surface, with some alteration from its natural colour. In one case only, a woman, aged 39 years, who had vomited, while in the hospital, a fluid sometimes brown, sometimes black; who had complete anorexia, laborious digestion, diarrhœa, and habitual cough, there were found, on dissection, ulcerations of the ileum, cœcum, and colon, and some tubercles in the lungs. The mucous membrane of the stomach was pale and blanched throughout, except near the great cul de sac, where there were three or four red spots, occasioned by capillary injection. All the coats of the stomach were otherwise apparently sound, though, but two days before her death, this woman vomited a large quantity of black matters.

Into the composition of the gastric parietes two layers of cellular membrane enter—one between the mucous membrane and the muscular coat—the other between this last and the peritoneum. Both of these are connected by means of cellular prolongations which pass through the interstices of the muscular fibres of the middle coat. These layers often become the seat of inflammation, and consequently changed in their structure. These membranes are rarely altered in texture by acute inflammation of the organ—and very often escape the effects of chronic phlogosis. At other times, these tissues undergo the same fate as contiguous parts, and become the seats of various changes of structure. There are cases in which these cellular layers are attenuated, and finally even disappear—in other instances, they become softened into a liquid pulp, and also disappear. In such conditions, the parietes of the stomach lose a great proportion of their natural strength, and are sometimes ruptured in the action of vomiting. But augmentation of thickness and density is the most common effect of chronic inflammation in these tissues, and this is the morbid anatomy of what is generally described as scirrhus of the stomach. In proportion as these effects take place, these cellular layers differ from their natural appearance, and present numerous and remarkable transformations. The most usual appearance is that of a grey, bluish, or dead white homogeneous membrane, without any trace of vascularity, and both dense and friable under the scalpel. This is called scirrhus, *par excellence*, and to this denomination there is no great objection, provided we bear in mind its real nature—namely, a thickening and induration of natural structure, devoid of any specific distinction from thickening and induration of the cellular membrane in any other part of the body. Thus, in a great number of chronic diarrhœas the submucous cellular membrane of the large intestines, becomes much more developed than in health—in fact, it becomes hard, white, and homogeneous. If this change be general in the said intestines, and to no great extent, it is called a thickening of the coats of the colon or rectum; but if it be partial, and to a greater extent of density and thickness, then it is called a scirrhous tumour, which is a palpable error—for there is no other difference between the two states than *degree*.

In some cases the induration of this tissue in the stomach goes on to an actual transformation into cartilage, of that soft and elastic kind, however, which we find in the fœtus.

If, instead of being white, homogeneous, and devoid of vascularity, it becomes full of vessels, it is no longer of a scirrhous nature, but takes the title of a cerebriform or encephaloid tissue, said to be developed in the parietes of the stomach. Formerly, our author admitted this distinction between scirrhous and cerebriform degenerations; but more minute investigations induce him to view them as only varieties of the same thing. He has seen them run into each other by insensible degrees, till all distinction was lost.

In these hypertrophied conditions of the cellular tissue of the stomach, we sometimes find cells or pouches, containing different kinds of fluid, varying in consistence and colour—sometimes resembling jelly—sometimes honey, &c. These have been called softened down scirrhi, but they are assertions without proof. These secretions are either infiltrated, as it were, through a certain extent of the cellular membrane, or more circumscribed, and, as it were, in cysts. If the activity of the vessels be considerable—if, in other words, the inflammatory action be in a certain degree, these cysts burst, and thus ulcerations are formed in the stomach, with discharges of puriform matter, or even fatal hæmorrhages. These secretions in the structure of the cellular membrane sometimes take on the tuberculous, sometimes the melanose character.

Hypertrophy of the gastric cellular membrane may be general, involving the whole parietes of the stomach, so that, when cut open, the organ does not collapse; but such is not a common event. Usually the hypertrophy is partial, and the pylorus is its ordinary seat. It is much more rarely found in the neighbourhood of the cardia. It seldom passes the limits of these openings to invade the structure of the duodenum or the œsophagus. In only two cases, our author has seen the disease spread to the lower third of the œsophagus, and there produce considerable stricture of the canal.

The muscular coat of the stomach generally escapes the disorganizing effects of chronic gastritis; but, occasionally, it becomes either hypertrophied, attenuated, or lost.

The blood-vessels also suffer considerable changes. Thus, beneath the softened mucous membrane, we often find very large veins, in a kind of varicose state. The same is seen in the neighbourhood of chronic ulcers of the lower extremities. This dilated state of the veins often continues long after inflammatory action has ceased, and, in several instances, our author found the parietes of the vessels themselves thickened by inflammation. M. Ribes has published some interesting observations on the part which phlebitis plays in certain cutaneous inflammations, and our author was led to investigate the same subject in regard to gastritis. The following phenomena presented themselves to him in two cases. On laying open the large and dilated veins which ramified under the mucous membrane, which was red and soft, in one case, brown and hypertrophied in the other, he ascertained that the venous parietes were thickened, and opposed considerable resistance to the scalpel, feeling hard to the touch, and not collapsing after being cut into. In the other case, where the stomach was the seat of a large ulceration surrounded with vegetations, the enlarged veins were seen in great numbers around the disease, and one in particular was remarkably enlarged and indurated, and the venules or radicals which led to it from the ulcer were also thickened and indurated in the parietes. On laying them open, they were found filled with a solid substance, of a dark red colour, mixed with a semi-liquid of a purulent character. The mouth of veins are not unfrequently found open at the bottoms of ulcerations in the stomach, although no hæmatemesis may have occurred during life.

The lymphatic system of the stomach and intestines does not always escape the effects of chronic inflammation. They have been found by our author filled with a puriform fluid—their parietes friable, and of tuberculous character—in some cases the parietes are thickened, and have lost their transparency.

The pathological condition of the nerves, in various diseases, has hitherto almost entirely evaded our researches, and there seems but little chance of morbid anatomy throwing much light on this department. Disordered function in the nervous system is attended with so small a proportion of change of structure, that in some of the most painful diseases, as tic douloureux, there is nothing to be found, after death, in the nerve, which was the seat of such excruciating torments during life. So it is with the stomach. M. Andral has dissected, with the greatest care, the nerves leading to and distributed on this organ, where the patients had died of painful gastric affections, of organic diseases of the stomach, and where there had been no disease or pain in the stomach—yet the nerves had the same appearance in all! The following reflections, however, are very just.

"Nevertheless we cannot, for a moment, doubt that there are alterations produced by diseases in the nerves, although we are, as yet, unable to appreciate them by the evidence of our senses. There can be no doubt that the various sympathetic affections of different parts of the system, dependent on disorder of the stomach, are produced through the agency of its nerves, so remarkable for their distribution and numerous connexions. Neither can we doubt that, among the various disturbances of function which the stomach undergoes, there are many which imitate, more or less completely, acute and chronic gastritis, but which are, in reality, owing to a morbid state of the gastric nerves or the semilunar ganglia—hence, in some individuals, we have disordered digestion, in others, vomitings, and, in others, epigastric tenderness, pain, &c."

Gastritis, whether acute or chronic, rarely terminates in gangrene. When it does so, it is generally where there have been ulcerations and fungosities, around which gangrenous spots occasionally shew themselves, and which take place when the powers of life are prostrated in the last stages of illness.

The stomach sometimes undergoes a change of volume, which is appreciable during life. The enlargement of cavity in a hollow organ is owing to various causes. Sometimes it takes place consecutively to an obstruction at the orifice of ejection, as we see in the heart, bladder, gall-bladder, &c. in which cases the parietes of the organ may remain of their natural thickness—be attenuated—or hypertrophied. At other times we find the dilatations take place in different organs, without any obstruction of the orifices whence their contents issue. This is especially the case with the stomach, which sometimes enlarges so as to almost fill the whole cavity of the abdomen, with or without any obstruction of the pyloric orifice. The folowing cases are curious.

*Case* 1. A woman, aged 65 years, meagre and miserable, began, about the month of June, 1821, to experience symptoms of an organic affection of the stomach, as difficult digestion, alternate anorexia and voracity, acid eructations, nausea, vomitings some hours after eating, pain in the epigastrium, &c. These symptoms increasing, she entered La Charité in March, 1822, being then reduced to a state of great emaciation. The space occupied by the stomach in the abdomen was well marked—indeed it was evident that it occupied the greater part of this division of the body. Its great curvature rested on the pubes, and the lesser curvature formed a sweep a little above the umbilicus. The patient had constant pain in what corresponded with the region of the pylorus, exasperated at intervals. She vomited a great quantity of black matters, and could not keep any food on her stomach. She died about a fortnight after entering the hospital.

*Dissection.* The thoracic and cranial organs sound. The abdomen being opened, the stomach was seen filling almost the whole of the cavity, descending to the left iliac fossa on one side, and stretching thence to the same situation in the other. The intestines were entirely concealed by the stomach, which contained an enormous quantity of dark-coloured fluid, resembling that which had been vomited. The mucous membrane was perfectly white, but much softened, and, for some inches round the pylorus, it was abraded, and an ulcer formed. No muscular fibres could be seen opposite the part where the mucous membrane was wanting. The orifice of the pylorus was not much narrowed—on the contrary, it was capable of admitting easily the point of the finger. There was nothing particular in the appearance of the intestines.

It is difficult to imagine the cause of this enormous distention of the stomach, without any material obstruction to the exit of its contents. Our author's explanation is far from being satisfactory, and, therefore, we shall omit it.

*Case* 2. A young woman, aged 23 years, had always enjoyed good health, till, having met with reverses of fortune, she repaired with her family to Paris, and there became a tutoress in a public school. Her health now soon began to suffer—her appetite failed—digestion became difficult, and, in February, 1821, vomitings were added to the other symptoms. Her food was not thrown up, however, till some hours after eating. She emaciated very fast. Leeches were repeatedly applied to the epigastrium, and various medicines were tried internally, but without avail. She entered LA CHARITÉ in February, 1822, in the last stage of marasmus. Her complexion was tinted—vomiting of food or drink took place almost immediately after swallowing them—slight pain in the epigastrium, which is soft, and presents no tumour or fulness—tongue natural—constipation habitual—pulse feeble—skin dry. She died on the 14th March.

*Dissection.* The stomach was dilated, and covered the greater number of the abdominal viscera. Its greater arch touched the pubes. The cavity of the organ was filled with a yellowish green liquid—the internal surface had a reddish or rosy tint, but in some places, especially near the splenic extremity, it was quite pale. There was a space, the size of a person's hand, where the mucous membrane was softened, and easily scraped off. The parietes of the stomach were, generally speaking, extenuated, and easily torn. The muscular tunic was remarkably wasted. The small intestines were much contracted, not larger than those of a dog, and their internal surface pale. There was some disease in the mucous membrane of the transverse arch of the colon. The liver was very much enlarged.

In this memoir, it will be observed that M. Andral attributes to chronic gastritis, several alterations of structure which usually have specific names, and are supposed to have specific characters—as scirrhus, melanosis, &c. These changes of structure have this one character in common, namely, that they are preceded or accompanied by various degrees of vascular activity, a circumstance which may be proved, or at least inferred, *first*, from the anatomical characters; *secondly*, from the symptoms during life, *3dly*, by the nature of the exciting causes, which give these diseases their development; *4thly*, by the treatment found to be most advantageous, namely, the antiphlogistic. But to say that all these diseases have one common character—sanguineous congestion—is only to discover a certain link by which they are connected—not the causes of their differences. How is it, it may well be asked, that, during the existence of this vascular activity, or sanguineous congestion, we see spring up such opposite kinds of changes of structure? M. Andral himself candidly acknowledges that we must take into account the predisposing causes, (or, in other words, the specific predispositions) in order to explain or reconcile such different results from the same inflammatory process—and that we must regard the sanguineous congestion as merely an occasional, or *exciting cause.*

"By the antiphlogistic treatment, unfortunately, we only combat this last, (the inflammatory or exciting cause) but make no impression on the specific predisposition, which, once excited into action, goes on to the formation of divers changes of structure, according to the nature of the predisposition. Hence the frequent inutility of blood-letting, which only attacks one of the elements of the disease. There are, it is true, cases where this sanguineous congestion plays a distinguished part; but there are others, where it appears quite a subordinate agent—and where, after a trifling *irritation*, we see produced the most formidable degenerations of structure in the stomach. There are cases, again, where this connecting link, or common feature, (sanguineous congestion) entirely disappears, and there is no proof that it ever existed. The disease should not then be called gastritis. Such are the different *neuroses* of the stomach—such are also certain cases of softening and extenuation of the coats of the stomach, which may be attributable to a real diminution of the act of nutrition, such as we see in the muscles of people who die of various chronic maladies." 193.

From this extract it will be seen that M. Andral modifies greatly the doctrine of inflammation, as applied to these different organic changes in the stomach, and that he views it as no more than a connecting link (and not always even that) which binds them in one genus, without at all accounting for their formation or their variety. "Pourvu que l'on ne regarde ce mot (inflammation) que comme indiquant *le lien* commun qui les unit mais nullement comme rendant raison de leur formation, comme pouvant expliquer leurs innombrables varietés."

We have placed this minute description of anatomical changes in the stomach on record in our Journal, because M. Andral is universally allowed to be one of the most accurate observers, and reporters of the present day. His opportunities for *post mortem* investigations in La Charité, are very great, and he turns them to good account. The document we consider as a very valuable one, especially to the pathologists of this country, where the means of research are limited, and where we are sorry to say the zeal for pathological enquiries is far below par. We will not lay the flattering unction to the souls of our countrymen upon this nor upon any other occasion, where the interests of science are at stake.

---

### 2. FEVER.*

Dr. Chambers has detailed some cases of fever occurring in St. George's Hospital, by which we are enabled to form some idea of this intelligent physician's practice, as well as doctrinal views of the disease.

Dr. C. is a warm advocate for the free use of calomel, assisted by other purgatives in the treatment of the common continued fever of this country. We cannot say, however, that our observations have led us to entertain quite such confident expectations from purgation in fever, as Dr. C. appears to have imbibed. "Those who have been in the habit of treating this disease, must have observed that, in most instances, when purgatives have been early and steadily administered, all the symptoms have, in a short time, yielded to them."

A case is first related by Dr. C. where a patient was admitted into the hospital, with the common symptoms of fever, where the tongue was of a bright red colour—the bowels irritable—the motions highly depraved,

* Dr. Chambers. St. George's Hospital. Med. and Physical Journal, October, 1826.

and the abdomen tense and painful. Leeches to the belly—five grains of calomel with antimonial powder at night—a rhubarb draught next morning—and the common saline effervescing medicine through the day. In a very few days of this treatment the patient was convalescent, and ordered to take bitters.

Dr. C. observes, however, that, notwithstanding the great advantages to be derived from this practice in general; still, some cases of fever will occur where, although the symptoms are mitigated, the patient continues to labour under the irritation of fever. "It is under these circumstances, that mercury, exhibited in small and repeated doses, appears to exert the most decided influence over the disease." What then are the circumstances or symptoms occurring in the progress of continued fever, which should induce the practitioner to change his mode of treatment, by abandoning the use of direct purgatives, and adopting the alterative practice? Dr. C. naturally conceives that, when this distinct remission or cessation of early symptoms has taken place, the primary fever is subdued—"and what remains of constitutional disturbance depends on those organic lesions which are now well ascertained to be ordinary consequences of continued fever." These lesions, in their turn, excite a new febrile action in the system, "which ought by no means to be considered as a relapse of the original disease." It is, in fact, a secondary fever, depending on irritation produced by structural injury; and is clearly distinguishable, Dr. C. thinks, from the primary disease, "by its remittent or hectic character," as well as by the extraordinary irritability, joined with prostration of strength, which accompanies it after it is established. The following case is offered in illustration.

*Case.* T. Kelly, aged 45, a labourer, was admitted on the 9th of August, 1826, the fever being of nine days standing. He had headache, fullness and pain in the abdomen, especially about the epigastrium, increased on pressure—pulse 110—skin hot. He was put on fever diet, which is chiefly farinaceous decoctions—to lose 12 ounces of blood, and to take five grains of calomel at night, with senna draught in the morning. In five days of this treatment, the local pains and uneasiness were gone, the pulse reduced to eighty, and soft. He only complains of debility. The calomel and purgative medicines were left off. The next day, the skin was dry and hot, the tongue furred, &c. Three grains of calomel were ordered with three of antimonial powder, every four hours. A blister to the nucha. He continued the mercurial, the quantity being gradually reduced, for ten days, by which time he had regained his natural manner, and every function was restored to a state of integrity.

Without, for a moment, questioning the principle which Dr. Chambers has laid down, we may be permitted to doubt, whether the foregoing case affords an illustration, or at all events, a proof of it. We see that on the 14th, all local pain and uneasiness had gone, and debility alone remained. The aperients being left off, he had camphor mixture and a few drops of spir. ammon. aromat. It is probable, though not stated, that at this period some allowance of food was made, beyond the *fever diet*. The very next day the skin was hot and tongue dry, &c. without any return of the local pain or uneasiness. Is it not probable that this relapse was occasioned by some little irregularity in diet, rather than by any "organic lesion" produced during the preceding attack? But Dr. Chambers shall speak for himself.

"It is not intended here to enter at length into the pathology of these changes. It will suffice to say that, when this cessation, or distinct mitigation, of the early symptoms of the disease takes place, it is probable that the primary fever has been subdued, and that what remains of constitutional disturbance depends on those organic lesions which are now well ascertained to be ordinary consequences of continued fever. These lesions, in their turn, excite a new febrile action in the system, which ought by no means to be considered as a relapse of the original disease. It is, in fact, a secondary fever, depending on the irritation produced by structural injury, and is clearly distinguishable from the primary disease by its remittent or hectic character, as well as by the extraordinary irritability, joined with prostration of strength, which accompanies it after it is established." *353.*

It happens occasionally that long before the primary disease has been overcome, the secondary irritation has commenced, so that, when the former is afterwards subdued, the latter presents itself to us in an aggravated form, and is, besides, the more intractable, because the strength of the patient is proportionally diminished. A case in illustration is added, for which we beg to refer to the Journal itself. We think the principle which Dr. C. has laid down is correct, and the practice judicious.

---

### 3. LIGATURE OF THE SUBCLAVIAN ARTERY. BY BARON DUPUYTREN.*

*Case.* Charles Lechevalier, 37 years of age, formerly a soldier, entered the Hotel Dieu, on the 27th February, 1819, for a false or consecutive aneurism of the left axillary artery. He had been wounded in the shoulder of that side, while serving in Spain, in the year 1811. The wound was made by a pointed instrument, but healed in three weeks. Two months afterwards, however, he perceived a small tumour in his arm-pit, attended with pulsation, and in the course of two years' captivity, it had acquired the size of an egg, and the pulsations were become very strong. After a long and toilsome march of 900 miles back to France, the tumour rapidly increased, and soon reached the size of a child's head, completely preventing him from following any occupation. On this account he came to Paris, and entered the Hotel Dieu.

The cicatrix of the original wound in the upper and posterior part of the shoulder was still visible. The aneurism was now the size of a

* Repertoire, No. 2, (Hotel Dieu.) Reported by M. Marx.

child's head of 12 months old. It was unequal on its surface, and covered with large veins, shining, tense, and throbbing in unison with the heart. There was numbness of this arm, and no pulsation at the wrist of that arm. The pulsations of the subclavian artery, however, were very strong, and pressure there stopped the beating in the tumour.

Nothing but the ligature presented any chance of saving this man's life, and the question arose, whether it should be applied *citra vel ultra tumorem?* Baron Dupuytren does not appear to entertain a favourable opinion of the method lately practised in this country by Mr. Wardrop.* The course of the subclavian artery on the left side may be divided into three portions—from its origin to its entrance between the scaleni muscles—its passage through these muscles—and its course to and over the first rib. The second portion of its course was selected by the Baron, for several reasons, among others, because the artery is here separated from the vein, and also from the plexus of nerves. Besides, the anterior pillar of the scaleni is a sure guide to the artery.

The patient being prepared for the operation, an incision was made in a slanting direction, an inch above the clavicle, through the integuments, by which some vessels were divided that required tying. The incision was then continued—the scalenus sought and found—and this muscle divided close to its insertion by a bistoury. The artery was thus easily laid bare, and its pulsations could be stopped by pressure of the finger at the bottom of the wound. A bent silver probe with an eye armed with a triple silk ligature, was passed under the artery, the other end brought out—the cord tied—and all flow of blood through the vessel arrested. The drawing of the ligature did not cause any pain in this operation, which circumstance is attributed, and probably with justice, to the situation which was selected for securing the artery. M. Dupuytren did not adopt the common continental practice, on this occasion, of placing a spare ligature ("Ligature d'attente") on the vessel. There was no hæmorrhage—the wound was simply dressed—and the patient put to bed. In the course of the day he complained of some pain in his throat, and he was bled by way of precaution. The night was tranquil—the arm preserved its temperature and usual sensibility—there were some dartings in the tumour. The second and third days were passed in the same favourable state, and the patient seemed as if he had not undergone an operation. At the end of six days, the tumour was considerably diminished, and the patient had experienced no inconvenience. He had had no motion the while, and a purgative was administered, (an ounce of castor oil,) which cleared the bowels. On the 11th day, the ligature came away, without any discharge of blood. By the 17th day the wound was contracting, the suppuration diminishing, the tumour was reduced one third, and offered no pulsation. By the 30th day the wound was cicatrized, the patient began to use his arm, the tumour was daily decreasing; but it presented

---

* *Apropos,* we wish Mr. W. would favour the public with the final resul of the operation on the old lady's carotid.

symptoms of suppuration. Cold astringent applications were used. By the 78th day the tumour was reduced to one-fifth its former volume, and was still getting smaller. All symptoms of suppuration had vanished. The heat, sensibility, and motility of the member were now nearly the same as in the other arm. The arteries at the wrist, however, as might be expected, presented no pulsation, although they felt full of blood.

The man was discharged from the hospital, and took to his usual occupations, which he pursued for three years, without the least inconvenience, till some heavy labour determined an inflammation in the armpit, for which he again entered the Hotel Dieu, on the 16th July, 1822. On examination, a tumour, the size of a man's fist, was found in the arm-pit, the integuments being inflamed and attenuated, and the swelling pointing and threatening to burst. It did not pulsate—the patient had had shiverings. Cataplasms were applied, and in a fortnight, it burst spontaneously and discharged a large quantity of matter of a peculiar kind. The symptoms now declined rapidly, and the man was soon discharged from the hospital perfectly cured. He has since remained well.

We cannot help expressing our conviction that the place which Dupuytren selected was the very best that could be pitched upon for securing the artery. When we read the descriptions of this operation, as heretofore practised—the diggings, the scoopings, the tearings, and the hæmorrhages, and compare all these with the simple and successful operation of the Parisian Cooper, we cannot withhold our meed of applause for the ingenuity with which the operation was devised, and the dexterity with which it was executed.

### 4. WOUND OF THE CAROTID ARTERY.—MR. TRAVERS, MR. FLEMING.

In the October number of the Medical and Physical Journal, Mr. Travers has related a case of wound of the external carotid artery, behind the angle of the jaw, for which a ligature was placed on the common trunk. The patient was a man of colour, strong, and about 35 years of age, who was brought to St. Thomas's Hospital, on the morning of June 27, 1826, with a small penetrating wound near the angle of the jaw, on the right side, apparently inflicted by a penknife while he was in a state of intoxication the preceding night. A surgeon who had been called, thrust a piece of sponge into the wound, and applied a bandage; the man was then conveyed to the hospital, and was in a state approaching to syncope, the extremities being cold, the face pale, pulse scarcely perceptible, intellects disturbed. On removing the dressings, a terrific hæmorrhage ensued, but was restrained by firm pressure. Mr. Travers having arrived, dilated the wound, removed a large coagulum, and endeavoured, but without success, to discover the bleeding orifice. Finding that pressure on the common carotid restrained the hæmorrhage, Mr. Travers resolved to tie that vessel. We need not describe this operation, as every pupil sees it often performed on the dead body. Immediately after tightening the ligature, a feeble pulsation was perceptible *beyond* it, as also a slight oozing of blood, both of which soon disappeared. The wound was then dressed, and the patient put to bed, with his hands secured, he being highly delirious. 28th. He is quiet and sensible, but morose; there has been very little hæmorrhage. Nothing particular occurred till the afternoon of the 29th, when a sudden excitement took place—the patient rushed from his bed, ascended the gallery of the operating theatre, and jumped from thence into the area, where he alighted on his feet—proceeded from thence to the great stair-case, and leaped over the balusters, from a height of 20 feet, alighting on a stone pavement, with no other injury than a slight bruise of one arm. He was seized and secured in bed. 30th. This morning he is again delirious, and succeeded in tearing off the dressings, but without doing mischief. July 2d. This morning he is delirious again. He was cupped on the temples, and had a large dose of calomel administered. 3d. The symptoms were:—countenance sunk, lips pallid, disposition to coma, pulse 72—wound healthy and granulating. 5th. A decided improvement. 9th. The ligature came away. There is still a wildness in his manner and countenance—wound filling by granulation—return of appetite. 12th. A paroxysm of delirium—appetite good. 15th. Had a cold shivering fit yesterday evening—granulations pale. 19th. Continues to improve. 27th. Ditto. Aug. 8th. Has been daily walking about since last report. Has had another slight shivering fit. The incision is healed, except about a quarter of an inch, whence a copious discharge issues. 18th. Hæmorrhage, twice, to the amount of eight ounces each time; which was restrained by pressure. 20th. Four hæmorrhages in the course of the day, but not to any great extent. 21st. Three bleedings. 22d. It is now necessary to keep up constant pressure. 23d. It was resolved to endeavour to find the bleeding vessel and secure it. In this attempt, at least two pints of florid blood, "in a full uninterrupted stream," were lost in less than two minutes. This hæmorrhage was repressed by the sponge and pressure—but the countenance assumed a ghastly hue, and in an hour and a half he expired.

*On dissection*, the cavity of an abscess was found, about the size of a pullet's egg, at the bottom of which was seen a portion of the entire cylinder of the carotid artery, about one-third of an inch in length, insulated, both above and below, by an intervening space of about one-third of an inch. The orifice of the lower portion of the carotid was circular, but its calibre diminished; and, on slitting it open, it was found that a plug of adhesive matter filled the vessel, and completely secured it against hæmorrhage. Nearer the heart was a long and firm coagulum of blood. The orifice of the upper part of the trunk was irregular from ulceration. It was from this part the hæmorrhage had proceeded, by reflux through the opposite carotids and vertebrals. On examining the artery at the site of the original wound, an aneurismal pouch, as large as a sparrow's egg, was found opposite the origin of the facial artery.

Before proceeding to Mr. Travers' remarks on this case, we shall take the opportunity of rescuing from oblivion a similar accident and a similar operation, the *latter* performed under more unfavourable circumstances, and yet with more fortunate results. The accident occurred in 1803, on board His Majesty's ship the Tonnant, of 80 guns, of which ship the late Mr. Fleming was then surgeon, and the present Dr. Coley, of Cheltenham, the assistant surgeon. The operation of tying the carotid artery was thus performed the year before Mr. Abernethy's case was published. The document is registered in the Archives of the Naval Medical Board of that period, and is authenticated by Dr. Coley, who accompanied the patient to Plymouth hospital, when cured of the wound and the operation.

"*Case.* On the 9th day of October, 1803, Mark Jackson, a servant on board His Majesty's ship Tonnant, attempted to commit suicide. When called to him, I found that he had bled most profusely from a large wound across the throat. The bleeding vessels were secured by ligature as quickly as possible; and when the blood was cleared away, I was enabled to ascertain the extent of the injury, and the critical situation of my patient. No pulse could be felt at the wrists; the pupils were dilated; the temperature of the body considerably diminished; and, in fact, he had the appearance of a corpse from which life had recently departed. The wound was about four inches in length, dividing the trachea, between the thyroid and cricoid cartilages, the two superior thyroid arteries, the internal jugular vein, and grazed the outer and muscular coats of the carotid artery. The mouths of all these vessels (except the last) had been tied in the first instance, and I now brought the divided parts, as well as I could, into contact, and kept them so by securing the head well forward on the chest.

"A small quantity of wine was got, with difficulty, into the stomach; and nearly two hours elapsed before he was sufficiently recovered to admit of being removed to the sick berth.

"On the following morning he was low and faint. There was considerable tumefaction of the parts, which prevented his swallowing; and nourishing glysters were therefore thrown up. In the course of the day the pulse could be felt at the wrists, beating feebly, about 100 in the minute. The wound put on a favourable appearance, and he enjoyed good nights. On the third day he was much troubled with a cough, which made me apprehensive of the rupture of the remaining coat of the artery; but all remained safe for the present, and the enemas were regularly administered.

"On the 15th of October, the ligatures came away, and the favourable appearance of the wound induced me to hope that all would terminate well. But the cough increased in violence, occasioned by the discharge gaining admittance into the trachea; and on the evening of the 17th, during a violent paroxysm of coughing, the artery burst, and my poor patient was, in an instant, deluged with blood! The spectacle was rendered still more horrid, by the danger of suffocation, and the torrents of blood that issued from the mouth and nose! Several attempts were made to secure the artery, but all failed, from the degree of ulceration that extended along the tube, and syncope alone rescued the man, for a time, from actual death. In this dreadful situation, I concluded that there was but one step to take, with any prospect of success; namely, to cut down upon, and tie the carotid artery below the wound. I had never heard of such an operation being performed; but conceived that its effects might be less formidable, in this case, than in a person not previously reduced by hæmorrhage. My resolution taken, there was no time to lose; and I therefore made an incision along the anterior edge of the sternal portion of the cleido-mastoideus muscle; then separating the parts below with my fingers and the handle of a scalpel, till I brought the artery fairly into view, I passed a ligature round it, and put an effectual stop to any farther loss of blood.

"I had now time to reflect on what I had done. The neck appeared, from the cut inflicted by the patient himself, and my dissection, one continued wound, and presented an alarming aspect. For some time, my patient lay as if deprived of life. When, by the aid of stimulants, he was a little revived, the edges of the wound were brought together in the usual manner. I did not expect to find him alive the next morning. He was so, however; though he scarcely appeared to exist. His pulse could with difficulty be distinguished; and when spoken to, he appeared much confused.

"The nutrient glysters were directed to be continued, and were all retained. His countenance gradually recovered animation; the heat of the body was slowly restored, and he passed quiet nights.

"On the 20th of October, his pulse was 90, with increase of energy; and he seemed perfectly sensible. It is here worthy of remark, that till this morning he had no evacuation from the bowels during the space of eleven days.

"On the 22d, the glysters were discontinued, as the power of deglutition was so far recovered that he could swallow wine and soup. The wound healed so rapidly, that on the 24th, the ligature was completely buried in sound granulations, and required to be removed by a cautious dissection. He had this day another evacuation, being the second since the operation.

"The remainder of the case may be summed up in a few words. His strength remained for a long time impaired, from the great loss of blood; but being well supplied with nourishing diet, he was discharged from the sick list on the 20th of December, 1803, and retained as an attendant on the sick till March 1804, when he was sent to Plymouth Hospital to be invalided."

This happened in 1803. In 1804, Mr. Abernethy's case was published in the Medico-Chirurgical Transactions, consequently the claim of priority is at least divided between Mr. Fleming and the latter gentleman. We can account for the case not having been published at the time.* Mr. Fleming sailed for the East Indies, shortly after the operation, where he soon after died. The Editor of this Journal recollects Mr. Fleming's mention of the case, when at Prince of Wales's Island, in the year 1805, and his intention to publish it on his return. He was doomed never to revisit his native shores, but friendship as well as justice demands that the operation should be recorded, as honorable to naval surgery. We believe that the above was the first instance in which the carotid artery was taken up, after a rupture or wound of the artery itself, and life preserved.

Mr. Travers observes that the case he has stated is valuable, as it establishes an important fact, "that ligature of the common carotid trunk is available to arrest hæmorrhage from a wound of the external carotid

---

* The case was published in the Monthly Series of the Medico-Chirurgical Journal for January 1817—exactly ten years ago, by Dr. Coley.

artery, so situated as not to admit of being included between two ligatures." The secondary hæmorrhage, after an interval of ten weeks, certainly does not impugn the accuracy of this statement, which is, indeed, verified, beyond all doubt, by Mr. Fleming's case. It is highly probable, as Mr. T. remarks, that, in the first of the above cases, had the operation of tying the carotid trunk been practised distinct and at a distance from the original wound, and had this remained undisturbed, the coagulum would have been gradually absorbed—suppuration would have been prevented—and the case would have terminated like that of aneurism, with the separation of the ligature. Mr. Travers does not appear to have been aware of Mr. Fleming's case, as he talks of the want of a precedent for the operation which he has performed. We can refer him to the Journal mentioned below, for the details of Mr. Fleming's case, as published by Dr. Coley.

---

### 5. INJURIES OF THE HEAD.*

*** æt. 30, received a kick from a horse on the head, Nov. 1st, 1825. On admission into the St. Louis, immediately after the accident, the following symptoms presented themselves. Insensibility; a transverse wound, three inches long, over the right eye-brow, with depression of the bone to the depth of an inch; the depressed portions were joined so exactly at an angle, that it was impossible to introduce an instrument between them capable of elevating them. *Venesection—sinapisms to the feet.* In an hour the patient recovered his sensibility and speech. *Nov.* 2. Some excitement, lids contracted, pupils sensible to the light. As there were no symptoms of compression, M. Richerand decided on not applying the trephine. All went on well till the 13th; the wound had nearly cicatrized, but the contraction of the eye-lids remained. Some degree of pain and stiffness of the lower jaw now took place, but, on the 15th, the man left the hospital, which, on the 1st December, he re-entered. The wound was then about an inch in length, the jaw could scarcely be opened, and the limbs were becoming rigid; this tetanic state made progress, and on the 15th he died.

*Dissection*, 24 *hours after Death.* The fragments of bone had pierced the dura mater, and were compressing the anterior lobe of the brain, which, at this part, was reddened and diffluent. This softening was greater in the cineritious than in the medullary substance, Arachnoid tunic, at the anterior and superior surface of the lobe, thickened, and covered with a sero-gelatinous layer of pus.

*Remarks.* Here the inflammation and disorganization of the brain seem obviously to depend upon the compression and irritation produced by the depressed fragmeuts of bone. In the next case, however, which we shall notice, the mischief was still more extensive, although the pieces of bone were removed.

---

* M. Paillard. Clinical Report from the St. Louis. Révue Med. Sept. 1826.

*Case* 2. * * * æt. 16, received a violent blow, Aug. 1st, 1825, on the left side of the frontal bone, near the eminentia, from a grinding stone, which broke in pieces. On his admission, directly after the accident, the bone was found fractured, and a splintered piece, the size of a five-shilling piece, driven into the brain. The fragments of bone were removed by means of the circular forceps, and some of the cineritious substance of the brain issued with them. Copious venæsection—and the wound was dressed simply. Insensibility. In the evening, another free bleeding. Next day, the patient was perfectly sensible, and could answer questions. Much soft bloody-looking cerebral matter issued from the wound—*venæsection.* The discharge from the wound now assumed a gangrenous appearance. The patient, on the fifth day from the accident, fell into a comatose state, but survived till the 26th, when he sunk.

*Dissection.* The fracture had extended no further than the wound. The tunica arachnoides covering the convexity of the right hemisphere was much inflamed, and covered with a layer of pus; this was the case, also, at the base of the brain. The left anterior lobe of the brain was almost entirely destroyed; what was left was in the suppurative state.

We shall now record another case, where fracture, with depression, is stated to have taken place, without any symptoms of compression supervening.

*Case* 3. Boulogne, æt. 22, a carpenter, of athletic constitution, fell on his head from a high scaffold, Sept. 1st, 1825. On admission into hospital, he was insensible; there was felt, over the right superciliary ridge, a depression about six lines deep, arising from the complete separation of the depressed portion of bone from the cranium. The integuments were bruised, but not broken. On being bled, the sensibility returned. *Sept.* 2. The man had a little pain in the head, and fever. V.S ad ℥xx. At the end of a month the patient was cured. The swelling of the integuments had subsided, but the depression still remained, about the depth of six lines.

To these cases we shall add one which occurred, some little time back, in a public hospital, and under our own observation.

W. S. æt. 12, of a strumous habit, fell from a scaffold, 60 feet high, on the 1st Sept. 1826. He was stunned by the fall, but was recovering, when a medical gentleman came up, and took away fourteen ounces of blood on the spot. On admission into hospital, he was quite sensible, and the pupils contracted and dilated. At the upper and left part of the head there was a scalp-wound, and the parietal bone beneath was found to be fractured into several portions, which were depressed. Cerebral matter issued through the wound. This was dressed with lint. For the three next days, there was little alteration—the pupils contracted and dilated, though not so readily as they should do; the lad was sensible, and could answer questions. *Sept.* 5. Re-action, in some degree, took place—the pulse got up—he would not answer questions—the pupils became more sluggish. V.S. ad ℥xij. with much relief to the symptoms. He soon, however, relapsed into his former dozing state—the pulse became 140 or upwards—convulsions appeared—the pupils became dilated, and delirium occurred at night. *Sept.* 8. The pupils again contracted more readily, but opisthotonos, chiefly, however, confined to the muscles of the back of the neck, was developed. *Sept.* 9. He gradually sunk. During the progress of these symptoms, pus and cerebral substance, blended together, were discharged pretty freely through the wound. The treatment consisted in frequent small bleedings, cupping between the scapulæ, and the exhibition of haustus sennæ and liquor antimonii tartarizati.

On dissection, the fracture was found to be chiefly in the left parietal bone, but extending into the right. The dura mater was lacerated to the extent of an inch or more, and beyond this laceration it adhered to the arachnoid. Below the laceration there was a cavity in the brain containing mingled pus and cerebrum in a diffluent state. Pus effused over the hemispheres of the cerebrum—pus and serum near the junction of the optic nerves.

We shall now make a few remarks on the subject of injuries of the head. Our impression decidedly is, that at this present moment, surgeons trust too much to nature, and too little to the instruments of their art. In Mr. Pott's time, almost every man who got a broken head was trepanned as a matter of course. This, no doubt, was bad practice. Mr. Abernethy published his work on injuries of the head, and the trephine was almost banished the surgeon's instrument-case. Indeed, Mr. Samuel Cooper, in treating of fractures of the cranium, with depression, again and again warns his readers—" that *existing symptoms of dangerous pressure on the brain can alone form a true reason for perforating the cranium.*" Surgeons are beginning to discover that this is bad practice also. If the bone is depressed, and there are as yet no symptoms, we are told that it is *too early* to apply the trephine, but when symptoms do come, sad experience tells us, alas! that it is *too late.* Now, when we consider that these *symptoms* are, for the most part, *not* the symptoms of mere compression, but of irritation and inflammation, excited by the presence of a foreign body, we should be led to expect their occurrence, not so much immediately on the receipt of the injury, as after the lapse of a certain period of time. And such, in by far the majority of instances, is the actual state of the case. We are convinced that the term *compression*, has done infinite mischief; it has led to false reasoning and consequent bad practice. Mr. S. Cooper, for instance, in his arguments against the use of the trephine, makes use of the following piece of sophistry: "indeed, it is not easy to conceive that the *pressure* which caused no ill effects at a time when the contents of the cranium filled its cavity completely, should *afterwards* prove injurious, when they have adapted themselves to its altered size and shape." *Page* 505. If it were always mere *pressure*, perhaps it would " not be easy to conceive it," but the cause of the mischief generally being *irritation*, it is not only " easy to conceive" the after occurrence of symptoms, but every one who is not wilfully blind may see the thing happening every day. What, then, is to be our rule in the application of the trephine? If the fracture, *with depression*, be a compound fracture, we should certainly say that the trephine or elevator is indicated, even though no symptoms are present. It may be true that some cases of this kind will do well without operations, but these are " rari nantes," and we do assert that no surgeon is justified in thus exposing his patient to the risk which he infallibly runs. The opinions of Sir A. Cooper on this subject, of whose experience it would be useless for us to say any thing, are, we think, so just, that we cannot avoid transcribing them here.

" If the fracture be compound, the treatment must be very different; because a compound fracture is followed, very generally, by inflammation of the brain; and it will be of little use to trephine when inflammation is once produced. It might be thought that it would be time enough to perform this operation when inflammation had appeared; but this is not the case; for if the inflammation comes on, the patient will generally die whether you trephine or not."—*Sir A. Cooper's Lectures, by Tyrrel,* vol. i. p. 305.

Sir A. Cooper's practice, when called to a case of this kind, is this: Whether symptoms of injured brain exist or not, he generally uses the elevator to raise the depressed bone, if possible. If the elevator will not do, the worthy Baronet applies the trephine, but this he uses "very rarely."

The case we have detailed as occurring at one of our hospitals illustrates well what we have said on this subject. A boy falls from a height of 60 feet, the scalp is torn—the bone depressed, and brain issues through the wound. The depressed bone is not elevated—the irritating body not removed. What is the consequence? Why all goes on well for a few days; then symptoms of irritation and inflammation shew themselves, and the patient sinks. On dissection, the membranes are found inflamed, and the brain ulcerated. Can there be a better case than this of the danger of *not* trephining?

There is, however, another kind of fracture, with depression, viz. that where the scalp is uninjured, and the fracture consequently simple, in which the use of the trephine may admit of doubt. Bearing in mind that the symptoms do not arise so much from compression as from irritation, it may, we repeat, admit of doubt, whether the conversion of the simple into a compound fracture, by the operation, and the consequent admission of atmospheric air into contact with the brain, may not prove a greater source of irritation than the mere presence of the sunken bone. Sir A. Cooper, for whose opinions we have the highest respect, observes—

" If the fracture be simple, and there is no wound in the scalp, and no symptom of injury to the brain, it would be wrong to make an incision into the part, and perform the operation of trephining; for, by making such an incision, you add greatly to the danger of the patient, as you make what was before a simple a compound fracture, and, consequently, greatly increase the danger of inflammation, *which rarely follows fracture with depression, where the fracture is simple.*" Even if, in this case, there *be* symptoms of injury to the brain, Sir A. Cooper would not immediately trepan, but would take away blood and purge freely, and then see how far the symptoms may arise from concussion of the brain, and not from depression.

Whether this be the best practice or no, we are not quite prepared to say. Case 3, which we have related in this paper, as far as it goes, confirms this view of the subject. The man had considerable depression of the frontal bone, but the scalp was sound. He had not a bad symptom. On the first kind of fracture, however, *compound*, with depression, we think there can be no manner of doubt as to the propriety, nay, the necessity of trephining. We cannot conclude without referring the reader to the very able paper on this subject, published by Mr. Wise, in the Extra Limites department of this Journal, for April, 1825.

### 6. OPHTHALMIA.*

Mr. Mackenzie, who is one of the surgeons to the Eye Infirmary of Glasgow, has published a paper on this subject, of which we shall give a short abstract. He divides the ophthalmia occurring in adults, from atmospheric causes, into the catarrhal, rheumatic, and catarrho-rheumatic. These are German distinctions, too little attended to, Mr. M. thinks, in this country, and essentially necessary for the proper treatment of the disease. "The appropriate treatment of the rheumatic ophthalmia is not at all adapted to the catarrhal; while the remedies which, in a few days, subdue the catarrhal, will only exasperate the rheumatic."

The catarrhal genus affects the conjunctiva—the rheumatic affects the "*fibrous sclerotica* and surrounding fibrous membranes"—the catarrho-rheumatic affects both the conjunctiva and the sclerotica, the symptoms being a union of those accompanying both. We fear Mr. M. will have some difficulty in persuading the routine practitioners of this country to adopt his minute classification. Thus in conjunctivity, as a genus, there are four species—the atmospherica—contagiosa—leucorrhoica—gonorrhoica.

The inflammation in the first species (catarrhal ophthalmia) which is the most common of all forms of ophthalmia, in adults, is almost entirely confined to the conjunctiva and meibomian follicles. The secretion of the membrane is increased, and becomes opake, thick, and puriform, though in many cases it remains translucid. The redness is chiefly in the conjunctiva lining the eye-lids, in mild cases, while the vessels on the white of the eye can be moved in every direction, by pressing the eyelid against the eye-ball with the finger, "shewing that they reside in the conjunctiva." In severe cases chemosis takes place, and general antiphlogistic treatment is insufficient—the cornea may burst, and vision be destroyed, if local means are neglected. Mr. M. attributes this accident more to mechanical pressure of the distended

* Mr. Mackenzie. Med. and Phys. Journal, No. 4.

conjunctiva of the eye-lids and eye-ball, than to excessive inflammation in the cornea itself. In this species, the patient uniformly complains of a feeling of sand in the eye, which may therefore be regarded as *diagnostic.* There is usually freedom from head-ache, which is the reverse in the rheumatic species, this last being accompanied by violent circum-orbital pain, aggravated in the night.

The exciting causes are atmospheric transitions, and especially exposure to the night air, to cold and to wet. The nature of the causes renders the disease sometimes epidemic. If neglected or improperly treated, it may continue for many weeks, and cause much febrile excitement, as well as local distress. The conjunctiva may become rough, and by its friction over the cornea cause nebulæ or even opacity. The discharge becomes puriform, and in that state will communicate conjunctivitis to others by actual contact, and in a still more severe and dangerous form than the original disease. Mr. M. thinks it probable that the Egyptian ophthalmia among our soldiers was at first "atmospheric puro-mucous conjunctivitis, but that it afterwards degenerated into a contagious, perhaps infectious disease."

Several examples of this change of character in the discharge are given. Indeed, direct experiments by Guillié, proving the contagious property of the matter.

The catarrhal ophthalmia yields readily, in general, to very simple treatment—"chiefly of a local and stimulating kind." Violent general remedies, Mr. M. thinks, are absurd, and worse than useless. We shall give the indications of treatment in Mr. M's own words, as we cannot abridge them without injury to the author's sentiments.

"1. I very rarely find it necessary to take away blood in catarrhal ophthalmia, either from a vein or by leeches. When there is more than usual constitutional irritation, the taking away of from twelve to twenty ounces of blood from the arm, will no doubt prove useful; but this will rarely be necessary, if the disease has not been neglected for a number of days, or mistreated.

"2. Scarification of the conjunctiva of the eyelids is necessary only in cases in which there is some degree of chemosis, and a distinctly puriform discharge. In such cases it proves a valuable means of cure. One or two deep incisions being made along the inner surface of the upper or lower eyelid, a very considerable discharge of blood will immediately take place; and, if the eyelid be properly managed, blood will continue to flow for a considerable time. For this purpose, the eyelid ought neither to be held everted till the bleeding ceases, nor allowed to fall back into continued contact with the eye-ball, in either of which cases it will soon cease; but the eyelid ought to be alternately everted and permitted to return to its natural position, by which means the divided vessels are refilled, and thus a continual flow of blood is produced.

"3. A brisk dose of calomel and jalap may be ordered, with occasional doses of neutral salts.

"4. Determining to the skin is also useful; which may be done by the warm pediluvium at bed-time, and by small doses of Spiritus Mindereri, or of any other mild diaphoretic, in combination with diluent drinks.

"5. In severe cases, a blister to the back of the neck will be found useful, or blisters behind the ears.

"6. Even weak solutions of acetate of lead, or of sulphate of zinc, are prejudicial in this disease, aggravating the sensation as if sand were in the eye, increasing the redness, and leading to opacities and ulcers of the cornea.

"7. On the contrary, the feeling of sand is uniformly relieved, and the inflammation abated, by the use of the solution of nitrate of silver. The solution which I employ contains from two to four grains of the nitrate in one ounce of distilled water. A large drop is to be applied to the eye once a-day, by means of a camel-hair pencil. The instant that it touches the eye, the salt is decomposed, and the silver precipitated over the conjunctiva in the state of muriate. I have sometimes alarmed other practitioners, by proposing to drop upon the surface of an eye highly vascular, affected with a feeling as if broken pieces of glass were rolling under the eyelids, and evidently secreting purulent matter, a solution of lunar caustic; and I have been not a little amused and pleased at their surprise, when next day they have found all the symptoms much abated by the use of this application.

"8. As a collyrium, I am in the habit of using a solution of one grain of corrosive sublimate in eight ounces of water. This being made milk-warm, is used thrice a-day for fomenting the eyelids, by means of a linen rag. In mild cases, a few drops are thus allowed to flow in upon the eye; but, in severe cases, in which the discharge is copious and puriform, this collyrium must be injected over the whole surface of the conjunctiva, and especially into the upper fold of that membrane, by means of a syringe; so that the whole morbid secretion is removed, and the diseased membrane immediately touched by the solution.

"9. At bed-time, about the size of a large pin-head of red precipitate ointment, melted on the end of the finger, is to be smeared along the edges of the eyelids. This ointment is prepared by levigating twelve grains of red precipitate till they become an orange-coloured impalpable powder, to which one ounce of fresh butter is to be added. I have occasionally seen this ointment prepared so carelessly, that crystalline scales of red precipitate were evident in it to the naked eye. The red precipitate ought to be carefully levigated till it lose the red colour, and become orange. Added to the quantity of unctuous substance above mentioned, it forms a golden-coloured ointment, which keeps for a great length of time, and is by far the best of all eye-salves.

"10. The inside of the upper eyelid ought daily to be inspected. If there is any tendency to a rough and sarcomatous state of the conjunctiva, it ought to be touched with the solid sulphate of copper." 326.

On this plan, our author has treated a number of cases of catarrhal ophthalmia, and with uniform success. In no case, if treated before ulceration took place, did ulcer or opacity succeed. On the other hand, he has seen many cases which had been much aggravated, by trusting to general treatment—especially to bleeding—or by the use of acetate of lead, or sulphate of zinc, as local applications. He has been led to attribute to these salts the detachment of the conjunctival layer of the cornea—whereas, such superficial ulcerations, treated with the solution of nitrate of silver, have uniformly healed without opacity.

An analogous mode of treatment is to be followed in the different species of puro-mucous conjunctivitis. But these are more severe and more dangerous diseases than the catarrhal. Mr. M. defers any farther remarks on these till a future opportunity. In the mean time he has introduced a number of highly interesting cases, in illustration of the principles of treatment which are laid down in this paper. For these we must refer the reader to the Journal of our contemporary already indicated.

---

### 7. MEMOIR ON MORBID INVAGINATIONS OF THE INTESTINES.*

[Dr. Dance—Hotel Dieu.]

Two cases which presented themselves at the Hotel Dieu, under M. Husson, suggested to his then pupil (Dr. Dance) the idea of this paper. He had some notion of writing an essay on the subject, enriched with researches among former writers; but has wisely come to the determination of doing little more than recording the facts, and there leaving them, as materials for future historians of a terrible disease, which is variable as to degree, obscure as to symptoms, and capable of destroying life without the practitioner being assured of the existence of the malady. Many cases of this affection are on record; but few of them, Dr. Dance observes, are sufficiently minute in their anatomical details. We now proceed to the facts, which form the basis of this Memoir.

*Case* 1. C. Claude, aged 35 years, a joiner, was admitted into the Hotel Dieu on the 19th April, 1825, presenting the character of long sufferings, and giving the following history of his complaint. Formerly a soldier, he had resided ten years in the neighbourhood of Rochefort, and had there been attacked several times with intermittents, for which he had taken bitters, emetics, and purgatives. For two years past he felt frequent dispositions to vomit, and then threw up bilious matters, especially after any errors in diet. During the last three months, this poor man was a martyr to vomitings, which occurred from hour to hour, and were sometimes accompanied by diarrhœa, in which blood appeared. His digestion was very difficult, and he had fallen into a kind of marasmus. His face was sallow and shrunk, with a yellow tint—skin dry and rough—tongue white in the middle, moist, and a little coloured at the sides—vomitings, especially after taking drink, abdomen retracted, hard and tense, pulse small and frequent, wandering pains in the belly. *Low diet—emulsions, &c.*

---

* Memoire sur les Invaginations Morbides des Intestins. Par M. Dance, M.D. Aide de Clinique à l'Hôtel Dieu. REPERTOIRE, No. 2.

20*th*. The diagnosis of "profound gastro-intestinal inflammation" was established. On the 21st and 22d, the vomitings were frequent, and the patient had constant desire to go to stool, without the power of evacuating any thing. This state continued. 23*d*. On examining his abdomen this day, there was observed, in the left iliac fossa, a kind of oblong tumour, the nature of which could not be ascertained. The patient had made more than twenty attempts in the course of the night to evacuate, but without success, having only passed some mucus and blood. He sunk this day, with all the symptoms of peritoneal inflammation.

*Dissection*. Great emaciation, and icteritious tinge of the skin. The head was not examined—the thoracic organs were sound. On opening the abdomen, the marks of peritonitis were every where conspicuous. The small intestines were distended with air. On wishing to begin the examination of the mucous membrane at the valve of the colon, they were surprised not to be able to find either cæcum or ascending portion of colon! After searching about, they came to a tumour which occupied the descending colon, and which proved to be an immense intus-susception, which was demonstrated to the pupils by Baron Dupuytren. The cæcum, ascending portion, and half of the transverse arch of the colon, appeared to be wanting, and the large intestine seemed to take its origin towards the termination of the transverse arch of the colon, presenting, for the space of 18 or 20 inches, an enlargement equal in size to that of a large adult arm, round, hard, and terminating abruptly in the left iliac fossa, where the colon resumed its natural form.

This tumour was formed by an invagination of the lower portion of the small intestine and a portion of the colon into itself. There were, therefore, three intestinal parietes to get through in proceeding from without to within—the first formed by the descending colon, which was the recipient of the whole—the second, by the ascending and transverse arches of the colon, incarcerated in the first, so that the cæcum was actually seated in the sigmoid flexure of the colon!—the third layer or coat of the tumour was the small intestine, which had descended as far as the new situation of the cæcum.

In the outer coat (colon) there were two large gangrenous perforations, one the size of the palm of the hand; the other rather smaller, and both penetrating through the three coats. The invaginated portions of intestine were glued together by adhesive inflammation, with false membranes, &c. between them. That portion of ileum which was nearest to the invagination was distended by fæcal matters and yellow fluids, and presented several erosions on its inner surface. On the other hand, that portion of colon which was below the intus-susception was shrivelled up, and contained only some mucus. The liver was much smaller and denser than natural, the other viscera healthy.

A question arises here, whether this remarkable invagination was of recent or ancient date. There can be no doubt but that it was of long standing, and that it had gradually gone to the extent above-described.

A very curious case of what was supposed to be invagination occurred, within these few years, in the person of a fine little boy, of ten or eleven years of age, residing in the New Road, and under the care of Mr. Bagster, an intelligent surgeon, of Guildford-street. The boy had been afflicted for some years with excruciating pain in the region of the transverse arch and descending colon, where, at length, a very tender and painful tumour was felt, and continued for more, we believe, than two years. All this time the boy was in the habit of passing, under excruciating torments, every two or three hours, some liquid excrement, mixed with mucus, blood, and serous fluids of different colours, but seldom, we believe, natural fæces. At length matters got to such a height that the boy was expected to die, and Sir Astley Cooper frequently saw the patient. On examination by the rectum, Sir Astley distinctly felt, we were informed, the invaginated portion of bowel, which had come down within reach of the finger. It was shortly after this period that we had opportunities of often seeing the boy. There was a hard tumour, exceedingly tense, tender, and painful, in the left side of the abdomen, apparently occupying the track of the descending arch and sigmoid flexure of the colon. The urgent desires to stool still continued, and the matters rendered were of a liquid and unnatural appearance, devoid of real fæcal smell. The boy was tolerably easy till the nisus for stool came on, when he roared out with pain, and, at these times, his mother pressed hard against the anus, which seemed to afford the boy relief. He was obliged to take opiates in considerable quantities, which, with aperients, were the only medicines that seemed to be of use. Externally, leeches were applied, from time to time, to keep down the inflammation. The most curious part of the history remains to be told. After so many years of suffering—after the existence of a tumour in an irritable or inflamed state for such a length of time—after an intus-susception had been ascertained by the finger of the first surgeon in England —after the boy's life had been long despaired of by friends and medical attendants;—he took a sudden turn for the better—the pains became less and less distressing—the tumour rapidly diminished, with a great discharge of fluid—the stools became more natural—and the boy, at this day, has not a vestige of disease left! It is worthy of remark, that, when the complaint began to get better, he felt something dragging up or retracting from the tumour, which is now supposed to have been the invaginated portion returning back again. We candidly confess that we did not consider this case as one of intus-susception when we first saw the boy. We looked upon the tumour as some morbid growth, probably an hydatid, which had burst into the colon. The termination of the case, however, has staggered us a little, and made us distrustful of our diagnosis. If it was an invagination, it offers a remarkable instance of cure by the effort of nature, after so many years' duration of a formidable disease.

*Case* 2. J. Pradier, aged 22 years, was received into the Hotel Dieu on the 8th August, 1825. He dated his complaint at several months previously—the symptoms being occasional colicky pains, inclination to vomit, and actual vomitings followed by constipation. These accidents, however, did not prevent him from following his usual occupations; till the end of May, 1825, when the sickness at stomach acquired a degree of intensity that compelled him to enter an hospital in the country, where leechings gave relief. He came to Paris in July, where he was soon so ill that he applied for reception at the Hôtel Dieu. He was now much emaciated, with pallor of face, expression of suffering in his countenance—colick and constipation—vomitings of greenish and inodorous fluids—severe pain in the line of the transverse arch of the colon, as also in the descending portion, extending to the anus, with a nisus for evacuation—tenderness of the abdomen on pressure—contraction and rigidity of the abdominal muscles—ardent thirst, but quick rejection of the fluids taken in—pulse small and hard—skin dry, but not hot. "All these symptoms appeared to be the result, solely, of a very acute peritonitis." We do not know how Dr. Dance and his master could reconcile this idea with the distinct pain following the line of the colon down to the anus. Such a phenomenon has no connexion with general peritonitis. Leeches were applied to the abdomen, and renewed the next day; to which were added half-baths and emollient cataplasms. 12th. The vomitings are more obstinate than ever—the pains in the abdomen more severe—and a tumefaction was remarked in the course of the sigmoid flexure of the colon. Some of the medical officers attributed this symptom to a collection of hardened fæces in the intestine; but the recent occurrence of the former case decided Messrs. Husson and Horteloup to consider the disease as intestinal invagination. (Thirty leeches to the abdomen, diluents.) 13th. No relief—vomiting, constipation, and tenesmus continue. A lavement of castor oil brought away some black and hardened fæces. 14*th.* Violent tenesmus—some bloody discharge. 15*th.* After a dreadful night of pain, the belly appears tumefied this morning—the countenance is unusually anxious—the eyes sunk—pulse small and frequent—abdomen painful on the slightest pressure. Sixty leeches. 18*th.* The skin this day became yellow—and death took place on the morning of the 19th.

*Dissection.* All the thoracic organs were sound. Head not examined. The peritoneum was universally inflamed, and formed numerous adhesions, by means of the false membranes thrown out among the convolutions of the intestines. On raising the small intestines, the cæcum and ascending colon were not to be seen; and in the left iliac fossa was found a large oblong tumour, ten inches in length, and as thick as a man's arm, its inferior extremity terminating in a conoid form, and of a dark brown colour. This extremity was formed by the mucous membrane of the cæcum inverted and invaginated in the sigmoid flexure of the colon, through the parietes of which it had burst, and projected into the pelvis. On examination, it was found that two inches of the ileum had descended into the cæcum, and this last had become inverted and pushed forward into the ascending colon, which, in its turn, had advanced into the transverse arch, and all these parts, thus invaginated, had gone down to the left iliac fossa. The surfaces were agglutinated together as in the former case. Three gangrenous perforations were discovered—one in the sigmoid flexure of the colon, already noticed—

N 2

the two others were in the parietes of the ascending and transverse arches of this intestine. All the coats of the intestines were thickened and discoloured. The small intestines, above the invagination, contained yellowish liquid matters, which became harder and drier as they ascended up towards the jejunum. There was no effusion in the cavity of the abdomen, nor any other disease than the above-mentioned.

We may now refer to some other cases of invagination recorded by authors. Baillie, in his Morbid Anatomy, speaks only in general terms of this accident, and observes that it is most liable to happen at the termination of the small in the large intestines. He does not state any case in that work.

The younger Monro, in his Pathology of the Intestinal Canal, relates the case of a child, four months old, to which he was called. The nurse informed him that the infant had had a copious evacuation that day, attended with great straining, since which he appeared to be in great pain. The pain, however, became mitigated, though the inclination to stool continued, with only the discharge of mucus and blood. Lavements would not go properly up, and purgatives did not pass downwards. Dr. M. suspected an intus-susception, and dissection proved the truth of his diagnosis. After 68 hours of misery the child died. On examination, it was found that the lower extremity of the ileum and the cæcum had been pushed up through the colon ascendant—afterwards through the transverse arch, and ultimately down to the rectum; that is to say—one half of the colon, including the cæcum and a portion of ileum, was reversed into the other half of the same bowel.

In the second volume of the Transactions of a Society for improving Medical and Chirurgical Knowledge, Dr. Baillie relates a case, which, however, he had no opportunity of seeing before death—the Doctor, at that period, (1795) being much more conversant with dead than living bodies. The morbid part was sent to Dr. B. by Sir W. Farquhar. The patient, it appeared, had been attacked with vomiting and violent pain in her stomach, attended with great constipation of the bowels. When motions were procured by strong medicines, they were mixed with blood and mucus. About three weeks before her death she voided a substance resembling gut, about a yard in length. For some days before and after this, she could not have an evacuation from the bowels "unless held up nearly in an erect posture." She could keep nothing on her stomach. Fourteen days before death the pain diminished, except in the left iliac region, where it still continued. Her body and legs swelled—any thing taken into the stomach immediately excited a disposition to stool, as well as vomiting. She died in convulsions after much suffering.

On close examination of the substance expelled, Dr. B. found it to be a portion of the colon. The statement is not very perspicuous, but the drawing which accompanies the paper, clearly proves the nature of the substance. No examination of the body would be permitted. There can be no doubt of the nature of the substance voided—and little of the modus in which it was separated. It is only an *invaginated* portion of intestine that could come away in this manner, without sudden death. The case related by Cheselden, of a poor woman who lived many years after the loss of 26 inches of gut that had become mortified in a hernia, is no objection to this conclusion, because the canal was preserved by the contiguity of the upper and lower extremities of the gut. A still stronger confirmation of our conclusion was afforded by a case published nearly a century ago in the Physical and Literary Essays, by Mr. Muir, of Glasgow. The patient was a boy, 12 years of age, who had been subject, for a year, to colick pains, diarrhœa, &c. and afterwards passed a substance which was proved by the elder Monro to be 13 inches of intestine. The boy died and was opened, when it was found that the portion discharged had been an invagination of the colon.

About 20 years ago, M. Roux read a paper in one of the medical societies of Paris, on a case of invagination of the colon, some account of which was published at the time in the 11th volume of the Medical and Physical Journal.

*Case.* A man, 48 years of age, was seized in the spring, with diarrhœa, after some excesses, attended with colicky pains. The complaint increased in severity, so that he was obliged to go from 20 to 30 times a-day to stool. During one of these efforts, a tumour appeared externally, the size of an egg, accompanied by violent pain. Attempts were made to reduce the tumour, but in vain. It increased in size. On the 3d day M. Roux and another surgeon were called, and found symptoms of abdominal inflammation, with extreme pain extending from the epigastric region down to the tumour, which had now a frightful appearance. It was about ten inches in length, and six or seven broad, of a chesnut colour, hard, and painful. On the 4th day there was hiccup. On the 5th day, the hiccup and pyrexia had increased, and vomiting was added to the other symptoms. On the 6th day, the vomiting was stercoraceous—the pulse small and weak—the tumour flaccid, with some diminution of volume. On the 7th day, all the symptoms were worse, though the tumour was more shrunk. The surgeons gave up the case for lost—and an old woman took charge of the patient. She managed to push up the tumour within the rectum. The man died next day. With great difficulty the surgeons got permission to open the body, when the following appearances presented themselves.

*Dissection.* On opening the abdomen much gas escaped, and there was a quart of dark-coloured fluid in the cavity. The intestines were coated with false membranes, and agglutinated every where to the parietes. The rectum, when opened, disclosed thirteen inches of colon invaginated. This formed the tumour which had come down per anum.

Dr. Shaw, of America, published, a good many years ago, a case of intus-susceptio, where the patient died after a few days illness, having first diarrhœa, then vomiting, with severe pain in the bowels, and a sense of their moving from side to side. On dissection, it was found that more than half the ileum had passed up into the jejunum, and was there invaginated. The whole of the small intestines were in a state of high inflammation.

Mr. Langstaff published, about 20 years ago, a curious case of intestinal invagination, of which we shall give an abridged account in this place. The patient was a fine healthy child (3 months old) who was suddenly seized, on the 10th of January, 1807, with griping pains in the bowels, violent vomiting, and frequent propensity to stool. Aperient medicines and a fomentation had been ordered by the apothecary. Next day, all the symptoms were aggravated, and nothing but blood and mucus came away. The sufferings of the child were excessive. A hard tumour could be felt in the left side of the abdomen, and the intestine protruded to some distance from the rectum, in consequence, as was supposed, of the straining. All the symptoms continued till the 15th January, when the child died.

*Dissection.* The cæcum, ascending, transverse, and descending portions of the colon appeared to be wanting. The ileum terminated in the sigmoid flexure of the colon, which felt externally like a solid body. The small intestines were distended with fluid excrementitious matters, and were highly inflamed. On opening the sigmoid flexure, the portions of intestine which appeared to be wanting, were here found, in an invaginated state. The invaginated intestine was highly inflamed, and its coats had given way in one part.

We need hardly remark that this case was almost precisely similar to those related by Dr. Dance.

Among the extraordinary efforts of Nature, in such a terrible disease, we may mention a case recorded in the 9th volume of Duncan's Medical Commentaries, by Mr. Dougall of Leith. A woman, 67 years of age, was seized with violent colic, "or ileus," and all the usual remedies were diligently employed for several days, without success. About the 10th day of the illness, a looseness came on, and continued four or five days with relief of the threatening symptoms. On the 17th day, she voided, *per anum*, a piece of the ileum, with its corresponding portion of mesentery, 18 inches in length. After this evacuation, she became almost free from pain, but was extremely weak. The woman lived till the 36th day—that is, 19 days after the discharge of the invaginated portion of intestine.

*Dissection.* The greater portion of the intestines was pressed down into the pelvis. On ripping open the small intestines with scissors, a part was found in a constricted state, where there had been an intussusception of the part thrown off. "There seemed evidently to have been a division of the gut, and an union of the two divided ends by inflammatory adhesion." The stricture at the union was capable of admitting only the little finger with difficulty. It would appear that this poor woman died rather of inanition than of the effect of the separation and discharge of intestine.

We shall now glance at a case, where Dame Nature was more successful in her efforts. It is related by the venerable Dr. Sanden of Chichester, in the 6th volume of Duncan's Annals. The patient was a young man, previously healthy, who was suddenly seized with violent pain in the bowels and sickness at stomach, on the 12th of May, 1800. The symptoms did not rapidly advance, as it was not till the 16th, that Dr. S. was desired to see the patient. He was then labouring under extreme pain, principally seated between the umbilicus and pubes, but extending down towards the right iliac region. The belly was tense—very tender to the touch—and increased in temperature. There had been stercoraceous vomiting—no passage downwards—pulse quick and hard—tongue dry—great prostration of strength—much restlessness. These symptoms continued nearly the same till the 18th, when the pain became much mitigated, and there was singultus. There was occasional vomiting of fæcal matters. The constipation still continued, though the abdominal tension had subsided. In the night between the 22d and 23d of the above month, a very copious and effectual evacuation by stool took place—and the next day, the patient complained for the first time, of great acidity in the matters ejected from the stomach. From this period all the symptoms appeared to recede, and the patient was soon convalescent. On the 27th, he passed by stool, a substance which, on careful examination, proved to be a portion of small intestine, measuring full twelve inches. Of this, the separation had taken place very obliquely at one end, and somewhat obliquely at the other; so that, in the part separated, the intestinal tube was complete for the space of only about five inches. To this a portion of mesentery was attached. The discharged portion was every where firm in texture, and only differed from its natural state in being of a darker colour.

In the first volume of the Transactions of a Society, &c. we have a paper on the subject of intus-susception from no less a personage than John Hunter. Mr. H. makes many just observations on the probable mode in which an intus-susception takes place. He then alludes to a case published by Mr. Whately in the 76th volume of the Philosophical Transactions, where the invagination began at the insertion of the ileum into the colon, carrying in with it the cæcum and its appendix. The ileum passed on into the colon, till the whole of the ascending colon, the transverse arch, and descending colon were carried into the sigmoid flexure and rectum. The valve of the colon being the leading part, it at last got as low as the anus; and when the person went to stool he only emptied the ileum, for one half of the large intestine being filled by the other, the ileum alone, which passed through the centre, discharged its contents. This appears to have been the case with the little patient of Mr. Bagster's attended by Sir Astley Cooper, and seen frequently by ourselves.

The case related by Mr. Hunter, was that of a child, nine months old, attended by Dr. Ash, who was seized with a strong spasm, not having any previous ailment, and during which, he passed a very large loose stool, followed by small mucous and bloody dejections. Dr. A. saw the child a few hours afterwards, and though apparently well, and taking the breast, he found the pulse very slow, which induced him to think mortification had taken place in the bowels. Purgatives, fomentations, the warm bath, and various glysters were employed, but without any good effect. Dr. A. felt, or thought he felt, a deep-seated fulness or hardness under the left hypochondrium. Blisters were applied. No evacuation by stool could be obtained, and the child sunk, 60 hours after the spasm.

*Dissection.* The small intestines were found much distended with fluid contents. The mesentery was so much confined, that the convolutions of the small intestines could not be readily followed. This confinement was discovered to arise from an intus-susception of the ileum and its mesentery, together with the cæcum, and ascending colon into the descending part of the sigmoid flexure of the colon; the mesentery of the ileum being drawn up so obliquely across the root of the mesentery, as to prevent the jejunum from having its usual freedom of attachment. The only part of the colon which could be seen, was the sigmoid flexure, in which was distinctly to be felt a hard substance consisting of the ileum and inverted colon. The sigmoid flexure being opened was discovered to contain the cæcum and colon in an inverted state. The inverted colon had drawn in the mesocolon and a portion of the omentum that was attached to the transverse arch. There was some inflammation and thickening of the contained colon. The sigmoid flexure, or containing part, had the natural appearance, except that it was dilated, and the other parts of the abdomen were in a natural state. The dissection appears to have been made by Mr. now Sir Everard Home.*

We have thus laid before our readers a mass of authenticated facts relative to this serious accident, which Dr. Dance has overlooked or did not think proper to adduce. These cases are quite sufficient to convey not only a clear idea of the nature of the disease, but of the places where it most frequently occurs, when of a dangerous or fatal nature. There can be little doubt that invaginations of the small intestines frequently occur, but only occasion a temporary inconvenience. We often see them in the intestines of children after death, and where they can be drawn out easily, exhibiting no sign of inflammation or adhesion. Helvin, in his celebrated memoir on gastrotomy for the relief of intus-susception, states on the authority of Louis, that among 300 children who died at the Salpetriere, during dentition or worm-fever, the greater number exhibited two, three, or four invaginations,

* Our readers will find, in the second volume of this Series, p. 460, a remarkable case recorded by Mr. Davies, where a child lived eight months with an intus-susception, and where the caput coli came down to the anus. The bowels were regularly open every day, the stools being thin and watery. In fact, there was no remora in the large intestines for the formation of natural stools, and a case of this kind is similar to one where an artificial anus is formed in any part of the abdominal parietes by a wound of the ileum. The fæces never touch the internal surface of the colon.

without inflammation, and without the children having shewn any symptom of such an accident before death.

But when invagination takes place at the valve of the colon—where the small intestine is dragged in, and the large inverted on itself, the case is otherwise, and the danger is imminent.

Several interesting questions present themselves to the mind, when reflecting on these accidents. The chief are—can we recognize them during life—and if recognizable, have we any, and what remedy for the accident?

In respect to the first question, John Hunter seems to answer in the negative. "An intus-susception, says he, can never be *perfectly* known till after death; but where there are violent affections of the bowels, attended with constipation, we have reason, from the cases which have been examined in the dead body, to suppose that this disease may be the cause of them. There are, however, so many other diseases which produce the same symptoms, that nothing can be ascertained."* It is evident that Mr. Hunter has been here rather too positive:—For surely the disease is recognizable when the invaginated portion is seen or felt at the anus, of which we have given examples. Again:—If, after the usual symptoms of invagination, a portion of intestine is thrown off, we have reason to conclude that an intus-susception had existed, even although the patient survives, and consequently where no dissection can be made. Of this, we have also adduced examples.

Dr. Hull, of Manchester, who has published some criticisms on the case stated by Dr. Baillie, (above detailed) is also of John Hunter's opinion, that there is no symptom or concourse of symptoms which will enable us to determine the presence, much less the seat of intestinal invagination, unless the part intus-suscepted come down into the rectum.†

Mr. Langstaff has ventured to criticise John Hunter's dogma (for which, by the bye, he was roughly treated by John Hunter's toad-eater, the late Dr. Adams) respecting the impossibility of our distinguishing the disease in the living body. "In many of the cases published, says Mr. Langstaff, and in that which is related in the present paper, *the seat of the disease was clearly denoted by a hard tumour in the left side of the abdomen.* This circumstance, together with the impossibility of throwing up more than a very small quantity of fluid by the way of glyster, and the presence of the other symptoms, *would lead us to suspect the nature of the disorder.*"‡

But it is to be remembered that, in many cases of invagination, the progress of the disease is slow, and consequently the symptoms must vary with the *degree* of the intus-susception. In the histories of cases, where dissection has ultimately *proved* the nature of the malady, we find that the early symptoms were, interruption, or at least embarrassment in the passage of air and fæces along the intestinal track; hence resulted nausea, hiccup, vomiting, constipation, frequent inclination to stool,

* Transactions of a Society, &c. vol. i. p. 112.
† Med. and Phys. Journal, Vol. 7, p. 36.
‡ Edinb. Med. and Surg. Journal, Vol. 3, p. 265.

colicky pains, exasperated by the least excess in regimen, imperfect digestion, flatulence, loss of flesh, &c. At this period of the disease, it is impossible even to suspect an intus-susception, because all the above symptoms are common to disordered states of the alimentary canal from various other causes, as chronic enteritis, stricture of the colon or rectum, &c. Even where the disease makes its debut with violence and abruptness, as in Dr. Sanden's case, for example, we can only conclude that the disease is an *obstruction* in some part of the canal, without being able to say what the *nature* of the obstruction is.

Dr. Dance, in the memoir under review, conceives that there is but one diagnostic sign on which reliance can be placed in these morbid invaginations—and that, when the intusception is in the large intestine. In the cases which he has related, and in those where dissection disclosed the real nature of the accident, there was not only a tumour, of greater or lesser dimensions in the left iliac region, but also a flattening in of the abdomen in the place usually occupied by the cæcum and ascending colon. These phenomena, in conjunction with the symptoms already described, can leave but little doubt, he thinks, of an intestinal invagination, seated in the colon. The hand accustomed to examine the abdomen, he remarks, will always be able to detect such a displacement of the contents, as exists in these cases. Collections of fæcal matters in the sigmoid flexure of the colon will be the most likely accidents to imitate these invaginations; but the history of the case and the symptoms will not, in all probability, be parallel in the two instances.

There are examples of invaginations of the ileum passing down into the cæcum, and there remaining without this latter intestine being moved out of its place. Here the symptoms will be very different from the cases mentioned above. The tumour in this case, will be in the right iliac region instead of the left. The impossibility of throwing up injections, as noticed by Monro and Langstaff, will aid in the diagnosis, where the invagination has advanced far in the colon.

The second question is one of great importance. Have we any remedy for these invaginations? Mr. Hunter observes that bleeding, to lessen the inflammation caused by the intus-susception, and to prevent the adhesive process by which the coats are glued together, as also "*quicksilver to remove the cause*," are the most obvious, and the means that are usually recommended. He remarks that, if the intus-susception be from below upwards, the quicksilver may get between the invaginated coats, into the angle of reflection, and by pushing it farther on, increase the disease. If the intus-susception be from above downwards, as is generally the case—indeed, always when the disease is situated in the colon, the quicksilver can do no good, as it must either make its way through the central aperture of the invagination—or, if stopped there, it can only increase the malady. Mr. Hunter, therefore, suggests that every thing which can increase the action of the intestine downwards is to be particularly avoided, as tending to increase the peristaltic motion of the outer containing gut, and thus to continue the disease. Medicine can never come in contact with the outer fold, and having passed the inner, can only act on the outer below, therefore cannot immediately affect that portion of the outer which contains the intus-susception.

"But we must suppose (says Mr. H.) that whatever affects, or comes in contact with the larger portion of the canal, so as to throw it into action, will also affect by sympathy any part that may escape such application. I should, therefore, advise giving vomits, with a view to invert the peristaltic motion of the containing gut, which will have a tendency to bring the intestines into their natural situation."

On this proposal Mr. Langstaff has made a most severe remark. "It must be a matter of regret that a name so universally and justly respected as that of Hunter, should be affixed to a proposal so manifestly absurd." We are not among the idolaters of John Hunter—but certainly John was no fool--and we are weak enough to discover nothing absurd in the proposal. Mr. L. says that vomiting is an early symptom of the disease, and consequently that to increase that symptom would be to increase the disease. Now we are of a very different opinion. We consider most of the symptoms of diseases as indications of the efforts of nature to remove them. If a person took arsenic, and vomiting followed, would Mr. L. consider an emetic as "manifestly absurd," because vomiting was an early symptom? If we could be certain that an invagination downwards had actually taken place, and that there had not been time for adhesive inflammation to glue the parts together, we know not a more likely means to retrovert the gut than the action of vomiting. The case which Mr. Hunter met with, was one of those sudden invaginations, where death speedily closes the scene; and really when we come to consider that Dr. Ash found the child apparently well, *after a strong spasm*, and then commenced *purgation*, because he suspected *mortification had taken place* in the bowels, from some peculiarity in the pulse, we are inclined to think that the purgation increased, if it did not produce the invagination; and that John Hunter's proposal was by far the more rational practice of the two. As to the second part of Mr. Hunter's therapeutical proposal, namely, the exhibition of purgatives, if the emetic did not succeed, (on the supposition that the invagination was upwards,) we think there is much doubt to be entertained on this point.

As for gastrotomy, it is scouted by Hevin, Hunter, Callesen, Hull, Langstaff, and the author of the memoir before us, Dr. Dance. But possibly the facts which have lately come before the public, respecting the comparative impunity with which the abdomen may be laid open, will alter our ideas a little on the subject of gastrotomy in ileus. Doubtless the adhesive inflammation which usually takes place in the course of the invagination, would render the operation generally abortive; but then we are to recollect that if the invagination were in such a condition as to render an operation useless, life itself will, almost to a certainty, be forfeited, and, under such circumstances, the *additional* danger of an operation is of very trifling consequence.

Some time ago,* we published the case of Dr. Fuschstius, as recorded

* Vol. III. of this Series (October 1825) page 537.

in Hufeland's Journal, where a man's abdomen was opened, after an intus-susception had continued for ten days, without any relief by stool. An invagination, two feet in length, was found, and with some difficulty extricated. The patient completely recovered. We then alluded to the case of Mrs. Belzoni's servant, where an obstruction remained obstinate for 10 or 12 days, when death took place. A piece of ileum was strangulated under an old adhesion, and was easily drawn out, without exhibiting any signs of inflammation. Gastrotomy would, in all probability, have saved this man's life. These considerations should induce us to pause before we condemn, without any qualification, the operation of gastrotomy in such dreadful cases. Anceps remedium melius est quam nullum.

One therapeutical indication, however, will be admitted by all parties. As it is evident that the adhesive inflammation of the parts will render the efforts of Nature and Art unavailing, if it goes to any extent, so the main point is to keep the circulation as low, by means of general and local bleeding, as is consistent with life. If agglutination can thus be prevented, there is still a chance of the invagination being set free by the efforts of Nature—and if these fail, an operation is much more likely to succeed, where all inflammation has been prevented by decisive depletion, than if the parts are allowed to become consolidated by effusions of coagulable lymph. We attribute the non-existence of intestinal inflammation in Mrs. Belzoni's servant, entirely to the daily bleedings, local and general, which were put in force. The physicians certainly did *their part* of the work in this melancholy case, and had Sir Anthony Carlisle screwed his courage to the sticking point, and performed gastrotomy, we should have been most happy to—

"Stick some sprigs of laurel round his wig."

Finally, let the surgeon bear in mind that the intus-susception, if it was such, in Mr. Bagster's little patient, disappeared after having existed *some years*—a consideration that may induce us not to conclude that adhesions must necessarily be insuperable, if the parts have been long invaginated.

Bleeding then, both local and general, should be carried, in all such cases, to the very utmost limit that is compatible with life—and nothing should be taken into the stomach that is capable of leaving any fæcal remains.

The forcible injection of water or air by the rectum, has been recommended. Mr. Langstaff objects to it, on the supposition that adhesion of the parts will prevent its success. But we are not to presume that adhesion has taken place, especially at an early period of the disease. Adhesion is the product of inflammation, which may or may not arise at a more or less early period, and which it is our business to keep down. Mr. Langstaff's objection, therefore, does not appear to be valid. We shall conclude with the following passage from Callisen, in the sentiments of which we entirely coincide. "Ilei *ab intus-susceptione* orti cura, si quæ datur, certé est difficillima, neque ulla alia methodus indicata videtur, quam quæ *inflammationi* et spasmo convenit."—*Syst. Chirurg. Part. poster.* p. 287.

P. S. We believe the late Talma died of this disease, and that Dupuytren would have attempted the operation of gastrotomy, had it not been for the debilitated state of the patient.

---

8. CHOREA. DR. M'ANDREW.*

It is now beginning to be acknowledged that Dr. Hamilton's plan of purgation will not always prove successful in chorea. That the disease depends upon irritation in the primæ viæ, in most instances, we have little doubt; but the causes of this irritation would not produce the disease, if there did not exist a morbid sensibility, or susceptibility, in the nerves of the alimentary canal. By purgation, therefore, we only perform half our work. We clear away all irritating matters; but we do not remove the morbid sensibility of the parts to which the irritants had been applied. On this account, the best practice is to follow up the purgative plan by tonics—or, at least, to combine or alternate them.

The case of Dr. M'Andrew was a girl, nine years of age, admitted to the South London Dispensary in March, 1826, her complaint having begun in January, in the form of slight convulsive movements in both sides of the body. These were always increased by mental agitation. She had occasional head-ach and diarrhœa. Her brother is subject to epilepsy. She herself had now the usual symptoms of chorea, but not in a severe degree. Purgation by calomel and jalap was assiduously employed, and leeches were applied to the head; but a month's treatment on this plan produced merely an increase of the convulsive movements. Æther, valerian, and opium were now conjoined with the purgatives, but under this treatment also the patient got worse. She could now scarcely stand or walk. The head was ordered to be shaved, and the tartar-emetic ointment applied, which brought out a copious crop of pustules. The convulsive movements on one side now subsided, The pustulation was extended to the spine, and with beneficial effects. The bowels were kept open by calomel and jalap. She ultimately, though very slowly, recovered.

It is evident that the purging plan failed here entirely, and the cure is to be attributed to the counter-irritation on the head and along the spine. This removed the morbid sensibility of the nerves, on which the disease generally depends. We repeat it, that purgation is only half the cure. Tonics, sedatives, and counter-irritants must be alternated or combined with purgatives.

---

9. M. LISFRANC'S CLINICAL REPORT.* LA PITIE.

1. *Carcinoma of the Eye.* We notice this more for the purpose of recording M. Lisfranc's mode of extirpating the organ of vision, than for any great interest that attaches to the case itself.

*Case.* Ann Vigé, æt. 21, of strumous habit, had suppression of the

* Med and Phys. Journal, No. 4, New Series.

* Hôpital de la Pitié. Révue Medicale, August, 1826.

menses at the age of seventeen, after which she was subject to ophthalmia of the left eye. In Nov. 1825, she received a blow on this organ, which caused much inflammation, and, indeed, deprived her of vision. On entering the hospital, on the 25th February, the eye was found to be irregular—the sclerotic of a deep livid hue—pus collected behind the transparent cornea, from which there arose a fungous projection—violent lancinating pains. Extirpation of the eye was determined on, and performed by M. Lisfranc, in the following manner.

The patient being seated, with her head reclining on the breast of an assistant, M. Lisfranc, placing the finger and thumb of the left hand on the external angle of the lids, drew out the integuments; then with the right hand introducing a straight bistoury horizontally beneath this angle, he passed it half an inch across, turned it forwards, brought the point out through the skin, and so made the instrument cut its way out. Then, with a hook, drawing out the globe of the eye, he plunged the bistoury between this and the walls of the orbit, on the inner and upper side, so as to divide the great oblique, and carried the instrument round to the point from which he started. It may be observed that the assistant opens the lids alternately, and not both at once, as is commonly done. The optic nerve and cellular tissue were divided by curved scissors, and the lacrymal gland cut out by the same instrument. M. Lisfranc, during the operation, avoided wounding the eye lids, by making the fore-finger of the left hand a director for the bistoury. Little blood was lost.

On the 5th day there was much pain in the head, with tumefaction of the palpebræ. These symptoms were dissipated by abstinence, a free bleeding, the application of 30 leeches to the temple, and a poultice to the part. The patient left the hospital on the 15th, quite cured.

2. *Hernia and Perforation of the Stomach, &c.* F. Duvenot, æt. 63, who had always been accustomed to carry heavy weights upon her stomach, had redness and pain about the navel, which, after a fall on the 20th March, 1826, became much aggravated, and ended in abscess, that burst spontaneously. On admission into hospital, April 4th, there was found, in the place of the navel, an abscess about the size of a five-franc piece, which discharged fetid matter. On introducing a probe, this was found to end in a cul-de-sac. The patient had spasms—the eyes were sunken—the extremities cold—tongue red and dry—pulse feeble and irregular. On the next day the discharge was more fetid, and smelt of fæcal matter, the probe passed to the depth of three inches. On the 12th, after the exhibition of a ptisan, pressure was made on the stomach, and the fluid was seen to issue through the opening. She sunk on the 18th.

*Dissection, 20 hours after Death.* The pylorus was found to have contracted adhesions with the umbilical tumour, and it had a perforation half an inch in diameter; the valve was almost entirely destroyed by ulceration. There was a very free communication between the stomach and duodenum. Ulcerations in the jejunum, ileum, and cæcum.

*Herpes Zoster.* In our last we took notice of the treatment of Herpes zoster by M. Geoffroy, at La Pitié, namely, cauterization by the nitrate of silver. M. Lisfranc has detailed three other cases in which this plan was tried, and with the effect of dissipating the eruption very rapidly.

3. *Wounds of the Scalp, with or without sub-aponeurotic Inflammation.* M. Lisfranc observes that, in scalp-wounds, unless the soft parts be very much injured indeed, the surgeon brings the divided edges together, and endeavours to obtain union by the first intention. Seeing, however, that in a great number of cases so treated, the union is only superficial, that purulent matter is collected at the bottom of the wound, and that depôts frequently form in the neighbouring tissue, giving rise to serious consequences, M. Lisfranc has adopted another, and, in his hands, more successful method. After cleansing the wound, &c. he brings the edges, not into close contact, but within two or three lines of each other, by adhesive straps, the interval is then lightly filled with charpie; over this there is put some common dressing, with holes for the free exit of matter, then charpie, and a bandage over all. If pain and tumefaction should occur, the dressings are removed, and merely a little charpie is introduced into the wound, which is covered by a poultice. He adduces several cases in illustration; we shall notice one or two.

*Case* 1. Gindon, æt, 32, robust, received a wound on the head, about two inches in extent, on the 9th January. He was bled, and the lips of the wound brought together, On the 11th he entered La Pitié. On the 12th the wound was found partly cicatrized, its edges tumid and painful; the pulse was frequent. On tearing off the cicatrix, a small quantity of matter escaped; the wound was kept open by lint and a poultice. On the 14th suppuration was established, and the wound dressed. On the 2nd February he was discharged, cured.

*Case* 2. M. Monty, æt. 67, received, Dec. 11th, a wound, two inches long, over the right parietal bone. On entering the hospital on the 12th, the pulse was quick and hard—intense cephalalgia. 13th, Venesection—the wound was dressed with charpie, simple dressing, and bandage. Jan. 16th. Discharged cured.

The practitioner, however, is not always fortunate enough to meet with cases thus early. Perhaps before he is called, extensive aponeurotic inflammation and suppuration have taken place, and an excitement of the system, which is, at all times, of a serious, and not unfrequently of a dangerous character. In cases of this kind, then, the cicatrix must be broken up, free vent given to the confined matter—poultices applied, and strict diet and antiphlogistic measures, if there is any determination to the stomach or any other organ, are to be employed.

*Case.* M. Débété, æt. 68, had a fall, in which he received a wound, two inches long, in the upper part of the scalp. On entering La Pitié, 4 days after the accident, the wound was found partly cicatrized—the edges tumid, œdematous, and of an erysipelatous redness—pressure gave great pain. Pulse quick—violent cephalalgia—extreme dejection. The cicatrix was torn up, and a poultice put on the part: bleeding, general and local, by leeches was employed, and, on the 10th September, the patient was well.

Several other cases follow, but they are of a precisely similar character, and to use the homely adage, "Enough is as good as a feast." We would now make a few remarks on the practice of which we have given an account. We think, then, that, with proper precautions, union by the first intention, in many cases of scalp wounds, as in other wounds, may be obtained with perfect safety to the patient. The error, in our opinion, in a great majority of instances, lies, not in endeavouring to produce union at *first*, but in continuing to force it on when the parts are manifestly not disposed to it. In scalp wounds where the vitality of the parts is not much injured, let union be attempted; if, however, there arise pain in the part, if the edges become puffy, and an œdematous condition shew itself around the wound, let not the surgeon, as is often the case, be continuing his straps and his compresses, but let the dressing be torn off, and a ready exit be given to the serum or the matter which is surely collecting. We say serum, for we have observed that a not uncommon consequence of scalp-wound, is an œdematous condition of the surrounding tissue, situated, for the most part, under the aponeurosis of the occipito-frontalis muscle; this œdematous condition of the cellular membrane between the occipito-frontalis and the pericranium is commonly attended with intense cephalalgia—great febrile action, and even delirium, and, if it be not relieved, it will, in all probability, end in extensive suppuration. A free incision down to the pericranium relieves the tension and mitigates the symptoms, and in one case, which occurred under Mr. Brodie, at St. George's, an erysipelatous attack, which appeared after the incision, was remarkably mild and speedy in its termination.

We conclude, then, that, whilst we do not entirely agree with M. Lisfranc, we think his plans, with the modification we have mentioned, very judicious, and we repeat, that the "adhesive" plan is often blindly followed up in this country.

### 10. DR. GREGORY ON VACCINATION.

[Small-pox Hospital.]

In the November Medical and Physical Journal, Dr. Gregory has made some remarks on the best mode of introducing the vaccine virus, in order to insure its effects. He thinks the most general cause of failure is the use of dry lymph on points or glasses. Another source of failure is an unfit lancet. This instrument should be clean and sharp, otherwise the virus is thrown back upon the shoulder of the instrument, and not a particle enters the wound. The skins of children differ much in the degree of toughness. Failures Dr. G. has observed to be more common where the skin is tough. It is desirable that the lancet should not only be extremely sharp, but have a broad shoulder. The skin should be put well on the stretch by grasping the arm firmly, and fixing the skin between the finger and thumb of the left hand. "In the hollow thus formed there is ample room for as many insertions as may be desirable." He thinks that six or eight punctures should be made, allowing them all to be effectual, in order to make the proper impression on the constitution. The form is thus :—

I I I
I I I
I I I

The lymph should be taken from a vesicle before it begins to subside. After the tenth day, the virus is scarcely fluid. It cannot be safely taken after the 8th day, including the day of insertion. We should say not after the sixth or seventh day. We generally take it on the fifth or sixth day. A fifth-day vesicle, he observes, will not generally afford virus for more than one subject. "An eighth-day vesicle (even when very tumid) cannot be relied on for more than six or seven subjects." The younger the lymph (fourth or fifth day) the greater is the degree of intensity. The propriety of inserting a numerous crop of punctures becomes very evident, Dr. G. observes, not only for the purpose of saturating the system more effectually, but for enabling the vaccinator of a public establishment to open a new vesicle for every third or fourth operation. The number of punctures here recommended will not add to the local inflammation, if they are made in the manner above described. The greatest number he has hitherto made was twenty; and, although the constitution sympathises more decidedly in such a case, the local irritation is not, *cæteris paribus*, greater here than under common circumstances. In a few cases he has observed a pretty copious eruption all over the body, of a lichenous character, disposed in crescentic forms, and receding in two or three days.

To ensure the success of the operation, the child should be in perfect health. It should never be vaccinated during hooping-cough. The vaccination *sometimes* fails, "from the prior occupation of the system by some other internal disorder." The most proper age, Dr. G. thinks, for the operation, is between the second and fifth month. If these instructions be attended to, our very intelligent author assures us that—

"Then the charm is firm and good."

---

### II. ANEURISM AT THE BEND OF THE ARM, OCCASIONED BY VENESECTION.

The first case of this kind which we shall notice is related by Mr. Dalziell, R.N. in the 4th Number of the Edinburgh Journal of Medical Science.

*Case* 1. In the early part of February, 1825, A. Craighton, æt. 57, a seaman, was bled by a surgeon in Lancaster, soon after which a tumour formed at the bend of the arm, and continued to increase. Compression was tried, but it gave so much pain that it was laid aside. On the 7th May, Mr. Dalziell, was called in, and found the arm swollen and œdematous; at its inside, near the tendinous aponeurosis of the biceps was a pulsating tumour, about the size of a lemon; much pain in the limb; pulse at the wrist small and weak. He was directed to live low, and the tumour was covered with a loose wet bandage. On the 10th the tumour was enlarged; the pulsation in it violent, that at the wrist weak. V.S. ad ℥xij.—purgatives and antimony. The skin now became thinner and discoloured, and, as he was evidently getting worse, the artery was tied on the 13th. It is unnecessary to detail minutely the steps of the operation; suffice it to say, that the vessel was dissected for about two inches lower than the insertion of the deltoid—that every care was taken to avoid the median nerve, &c. that two ligatures were applied, and the artery divided between them, and that, immediately upon the tightening of the threads, the pulsation in the tumour ceased. The wound healed readily; the pulse at the wrist on the 25th was distinct; that in the tumour disappeared; the tumour itself decreased in size, and, on the 29th June, he was quite recovered. Dec. 2. He had been at sea and remained well. The swelling was no bigger than a hazel nut, and he could move his arm freely.

*Case* 2. This occurred lately at St. George's Hospital. The patient, James Rogers, æt. 42, a butler, was bled, on the 30th of August, by a surgeon, who wounded the brachial artery. The bleeding was checked by a very tight compress, and the tourniquet. On his admission, Sept. 1st, the limb was much swollen from the extravasated blood, it was very painful, and a large, ill-defined, pulsating tumour was seen at the bend of the arm.

At 1 o'clock, 36 hours after the accident, the brachial artery was tied by Mr. Brodie, both above and below the puncture, though with some difficulty, in consequence of the displacement of the biceps muscle, &c. by the blood extravasated around. As there was little chance of union by the first intention, the wound was kept open with lint. Next day there was a good deal of irritation, both of the wound and of the system in general. On removing the dressings, much yellowish matter was discharged, and the relief was immediate. The bowels were kept open by senna and jalap, and ten grains of pil. opii were given at bed-time.

On the 11th one of the ligatures came away, and, on the 13th, the other followed. Some degree of tumefaction and pain appeared at the inside of the arm, giving rise to the suspicion that matter was forming there—these symptoms, however, in a few days disappeared. On the 26th an erysipelatous blush appeared on the arm, and spread to the fore-arm, attended with constitutional disturbance, and much pain in the direction of the radial artery at the wrist. By the 30th, however, this had quite gone off. The surface of the wound was granulating, though rather indolently—the hand was not numb, and he had some power over the fingers—there was no pulse at the wrist, nor, indeed, had there been any since the operation, and, as the patient was anxious to get into the country, he was dismissed the hospital.

*Remarks.* This case is one of diffused aneurism; if the case had run on, a sac would most probably have been formed by the cellular membrane, and the aneurism would have become circumscribed. The treatment, both as to the operation itself and its *sequelæ*, we think highly judicious.

In the Lancet, for September the 9th, there is a report of a case of this kind, treated at the Westminster Hospital, and a curious report, in good truth, it is.

*Case* 3. A man, æt. 35, was bled, July 30th, by a druggist, who punctured the *radial* (?) artery. Hæmorrhage, more or less, occurred every day, and he was admitted into hospital on the 4th of August, when Mr. White "cut down upon the tumour," (what kind of tumour?) "and secured the *radial* artery above and below the puncture." *Aug*. 10th. Hæmorrhage from a vessel in the wound: this was secured, and the hæmorrhage ceased. The wound now became unhealthy, and the tendon of the biceps and [illegible] of the pronator teres were exposed by ulceration. *Aug*. 22. Hæmorrhage, which, after the loss of thirty ounces, was stopped by the house-surgeon's compressing the brachial artery until the arrival of Mr. Lynn, Jun. who tied that vessel two inches above the upper edge of the old wound. *Aug*. 23. Pulse "a mere thread;" wound sloughy. A consultation was held between Mr. Guthrie, Mr. Lynn, Jun. and Sir A. Carlisle, who observed that, if the hæmorrhage returned, amputation must not be thought of, as the patient had lost so much blood that he would, in all probability, die upon the table! It was accordingly decided on tying the brachial artery higher up, or even the axillary, if the bleeding should return. *Aug* 24. Bleeding again. Mr. White then amputated the limb just above where Mr. Lynn had applied the ligature. The report leaves the man convalescing, but we learn that he has long since bid the world good night.

On dissecting the limb there was found a large trunk at the back of the arm; the profunda superior was found *unusually* large; so were its radial and ulnar branches, "affording an easy explanation of the cause of the repeated hæmorrhage, viz. the great supply of blood sent by these vessels into the anastomosing branches round the condyles." *No high bifurcation mentioned.*

*Remarks*. This is a good sample of a Lancet report. Our readers are, no doubt, aware that the consequences of wounding the artery in phlebotomy are fourfold, namely, diffused aneurism—circumscribed aneurism—aneurismal varyx, where the artery communicates directly with the vein—and varicose aneurism, where there is an intermediate sac between the artery and vein, which still communicate with each other. In the two last no operation is required, unless, indeed, in a rare case or two of the *varicose aneurism*, where the sac has so extended itself as to compress the vein, and form a common aneurismal tumour. Now which of these four accidents happened in this case? The reporter merely says, "Mr. White cut down upon the tumour;" but what kind of tumour that was, no one, from the description of the case, can possibly discover. To be sure the scribe, in his *remarks (!)* rates Mr. White for cutting down upon "a varicose aneurism," (which, elsewhere, he calls an aneurismal varyx, two different affections,) but we could almost stake our existence that he does not rightly know what a varicose aneurism is.†

Then, again, the case is introduced as an aneurism of the *radial* artery, but unless there was "a high division," (and not a syllable is said about that) how came the radial artery in the way? We suppose the *brachial* artery was meant; however, it matters not to him of the Lancet.

The account of the dissection is quite equal to that of the symptoms. Many surgeons, we believe, have an opinion, that, if a main arterial trunk be tied, it is not an uncommon thing for the collateral vessels to become, in a short time, very much enlarged. Our *intelligent* reporter, however, appears to be much struck by this singular circumstance, and ingeniously remarks, that it affords "an easy explanation of the cause of the repeated hæmorrhages." An important piece of information this!

After this, no one, of course, will think of reading the partial and inaccurate reports of the surgeons themselves; nor will any one be hardy enough to question the accuracy and talent displayed in the hospital cases of the Lancet. No wonder the latter is furious at the bare thought of "youths of 15 or 16" being obliged by "the tyrannical junto of Lincoln's Inn" to attend some lectures before they walk the hospitals. Why, certes, if this odious and *absurd* law were put in force, the publication in question would be robbed of half those happy illustrations of hospital surgery which now form such an ornament to its columns. No! let the "youth of 15" *walk* the hospital, and furnish as many cases as he will to the Lancet, without being compelled to the iniquitous drudgery of previously learning something of the principles of the practice which he records.

Men of Lincoln's-Inn, look to this.

---

### 12. ON THE NATURAL CURE OF WOUNDS OF THE INTESTINES.*

M. Paillard, Member of the Athénée de Médecine, has read to that body a memoir on the natural cure of intestinal wounds, which may help to explain some of the anomalies which not unfrequently occur in practice.

He observes that the intestine may either be simply punctured, or it may receive a longitudinal or a transverse wound, varying, of course, in extent.

If it be a simple puncture, the muscular coat contracts, and the mucous membrane is puffed up, (boursoufflé) so as to prevent the escape of solid or liquid matters from the opening. Gas may get abroad into the abdominal cavity, but the puncture cicatrizes, and prevents the exit of more, whilst that which has escaped is soon absorbed. Cicatrization,

---

† We have, since this was written, made enquiries on the subject, and we learn, what we expected, that the case was one of DIFFUSED ANEURISM!!

* Hop. St. Louis.

then, is one mode of reparation, but another, and, we should say, a more frequent one, is the supervention of inflammation, exudation of coagulable lymph, and adhesion of the punctured part to the peritoneum, epiploon or peritoneal covering of some neighbouring viscus. If it be a longitudinal wound, the same inflammatory action, and the same adhesion to the parts in contact with it, form the natural cure. If the longitudinal wound be very extensive, it often happens that the omentum falls upon it, gets between its edges, contracts adhesions, and thus forms a natural plug, preventing the effusion of the contents of the intestine, within the cavity of which it hangs, and is soon covered by an organized mucous membrane. It is thus, too, that the transverse wounds are closed. But if, as, alas! sometimes happen, the intestine be entirely divided, we dare not hope for this sanative process. In that case, the ends of the gut retract and contract, allowing, at first, only the issue of gas—but soon they relax, the matters they contain are discharged into the cavity of the abdomen, and death, of course, ensues.

One of the most characteristic signs of wounded intestine, whether it is cleft by a sabre, or ruptured by a blow, &c. is sudden tympanitis.

Our author adduces two cases in illustration, which occurred in the wards of the Hôpital Saint Louis.

*Case* 1. N. æt. 22, was thrown down by a voiture, the wheel of which passed over his belly. On being carried to the hospital, there was found no lesion of the walls of the abdomen—no pain—belly blown up, and sounding like a drum. He was bled several times, and numerous leeches and cataplasms applied to the abdomen. He convalesced quickly, but an unexpected hæmoptysis occurred, which carried him off two months after his admission.

*Dissection.* The small intestine, at one point, adhered to the peritoneum and last false rib, as well as to the omentum, which lay before it. On examining the interior of the gut, there was found a kind of loose plug, which proved to be the epiploon, inclosed in an opening, nearly four lines in diameter.

*Case* 2. N. æt. 50, was brought to the Hôpital Saint Louis, in consequence of a beam having fallen with violence on his belly. There was great tympanitis, but little pain. He died the same night.

*Dissection 24 hours after Death.* The small intestine was entirely divided; so was the mesentery, to the extent of about six lines. The two ends of the gut had separated, and were so contracted as scarcely to allow the introduction of the finger. Stercoraceous matter was found in the cavity of the abdomen.

*Remarks.* We have thus given a condensed account of M. Paillard's paper, and of his views;—views partly the result of experiments on animals, and partly of clinical observation in the Hôpital Saint Louis, of which he was an "éléve interne." The whole forms a sort of corollary to the work which Mr. Travers has published on "Wounds of the Intestines." It appears in these, as in other cases, that the safety of the patient depends on the supervention of the adhesive inflammation, and this will account for the comparatively greater danger of small punctured, than larger incised wounds. In the former case, the immediate injury to the part is trifling, the call for the reparatory or inflammatory process, therefore, less urgent, and the consequence is, that adhesion often does not occur. In the latter case, an active inflammatory state is at once superinduced, and the chances of adhesion proportionately great.—*N Bibliothèque, Juillet,* 1826.

---

### 13. DR. HEWETT ON FEVER.*

In our tenth Number, page 457, *et seq.* we gave some account of Dr. Hewett's paper on follicular ulceration. The present contribution contains a series of cases treated at St. George's Hospital, upon the principles laid down in his preceding papers. The treatment hinged almost entirely on local depletion, by leeches, from the abdomen, and the exhibition of aperient medicine, generally of the mercurial kind. Mercurial alteratives were also usually administered. When the patients entered the hospital, the period for active purgation (a practice much recommended by Dr. Hewett) had passed, and, therefore, lenient measures only could be employed.

In Dr. Hewett's second paper, in our contemporary, the ingenious author has endeavoured to ascertain the period of the fever, at which the follicular ulcerations become established; but this paper we are unable to analyze as fully as we could wish. His plan of treatment varies necessarily with the stages of the disease. These he divides into three—the *first* comprising the period between the incipient and the complete development of the mucous follicles, prior to any inflammation or erosion of the neighbouring membrane. The *second*, the period of the incipient and advancing ulceration of the mucous follicles, generally accompanied by some slight inflammation of the neighbouring exhalent surface. The *third* embraces the period of the separation, by sloughing, of the previously disorganized follicles, with their neighbouring structures, and the termination of the ulcers, either in cicatrization, or in the death of the patient. The plan of treatment for the first period is "the effective exhibition of calomel, combined with any other auxiliary purgative and sudorific." In three or four days of this treatment the abdomen generally becomes less hard and prominent; but the purgation should not be left off till the belly feels soft and supple. This system being followed up for five or six days, and the evacuations still continuing unhealthy, Dr. Hewett very judiciously advises the purgation to be slackened for a day or two, and a dose of castor oil given, "for the purpose of ascertaining whether the unhealthy character of the evacuations may not have been prolonged by the continued irritation of the purgatives." He has observed that castor oil, in such circumstances,

---

* Med. and Phys. Journal, Nov. 1826.

will often bring away natural, though liquid motions, and produce a subsidence of the abdomen.

Dr. Hewett has criticised the therapeutics of the disciples of Broussais, while he acknowledges the merit of their pathological labours.

The second stage of the follicular disease is to be chiefly combated by repeated applications of leeches to the abdomen, while two or three grains of calomel, and the same quantity of James's powder, may be given every six hours, exhibiting, occasionally, small doses of castor oil. If the bowels are already relaxed, a quarter of a grain of opium, or three grains of extract of white poppy may be added to the pills. The same measures may be pursued even after follicular ulceration has been established, if the symptoms indicate local inflammation and febrile excitement.

These three papers of Dr. Hewett will be read with great advantage by the profession in the pages of the medical journal in which they are published, and to this source we refer our readers for further particulars.

---

### 14. MR. EARLE ON LOCAL IRRITATION.*

The "constitutional origin of local diseases" has become so prevailing as greatly to obscure the "local origin of constitutional diseases." The former is the fashionable doctrine in England—the latter in France. Truth lies between; and we are much inclined to think that, as knowledge increases, the local seats of what are considered general diseases will become more conspicuous. The case related by Mr. Earle is a very curious example of the influence of a local irritation over the constitution at large.

A man had been wounded by a musket bullet in the East Indies, so long back as the year 1822. The thigh bone had been fractured, but the ball was removed, and all went on well for six weeks, when the fracture was displaced. After this, repeated abscesses formed, accompanied by much constitutional disturbance. There was a large cicatrix in the place where the ball had entered, and, in its middle, the opening of a fistula, leading to the bone. The bone itself was enlarged. Issues were made in the neighbourhood of the supposed diseased bone, and the general health strictly attended to. The fistulous opening frequently closed, and then opened again; and, during the formation of these abscesses, his health suffered severely, and he had many violent rigors. Acute rheumatism then attacked him in the joints, and afterwards retroceded to the chest and diaphragm, requiring active measures. Then he became affected with a well-marked intermittent fever, which was relieved by bark and ammonia. Worn out by these repeated constitutional attacks, the poor man requested to have his limb amputated. But Mr. Earle, conceiving that there might be some foreign body lodged in the neighbouring parts, keeping up irritation, examined the limb with great attention, and, on discovering a suspicious point, he there made an incision, and detected a large portion of dead bone, quite

* Med. and Phys. Journal, No. 5, New Series.

loose. This was readily extracted, together with two smaller pieces. They were fragments of the femur, being quite bleached and inodorous. From the very day after the operation, all constitutional disturbance ceased—the man rapidly regained his health and spirits—and was discharged quite well.

We agree with Mr. Earle, that the above is a striking example of the effect of local irritation on the constitution, and one in which the connexion between cause and effect is unequivocally established.

---

### 15. DRS. HAWKINS AND CHAMBERS ON PURPURA HÆMORRHAGICA.*

[Middlesex Hospital.]

Dr. Hawkins has detailed two cases of this curious disease. The first was a girl, 14 years of age, who came into the hospital covered with petechiæ, of a red colour, interspersed with larger, irregular, and purple ecchymoses. About a week previously, an oozing of blood had commenced from the mouth and nostrils, and the evacuations were dark, but not bloody. Her pulse was 140, skin cold, lips and tongue covered with black sordes, countenance very pale. The plan of treatment was purgation, principally by means of calomel and jalap, taking the acidulated infusion of roses, with sulphate of magnesia, and, towards the commencement of convalescence, some tincture of steel and digitalis. In three weeks she was well.

The second case was that of a boy, 14 years of age, who had been ill, with dysenteric symptoms, before he came into the hospital. Leeches were applied to the abdomen, and he had calomel and opium till the stools became of natural consistence, but they were still attended with discharges of blood. It was now observed that he had numerous petechiæ on his legs and arms, and his debility was extreme. An infusion of mint and cloves, acidulated with sulphuric acid. In a few days the intestinal hæmorrhage ceased; the petechiæ and vibices disappeared, and the strength began to return. In less than a fortnight he was discharged convalescent.

Dr. Hawkins has appended some remarks on this disease generally, which, he seems to think, is seldom dependent on increased force of the circulation, the vessels themselves remaining healthy. It appears that he is inclined to refer all cases of this disease to either a preternatural weakness of the capillaries, without increase of impetus, or a concurrence of increased impetus with vascular weakness. Immediately after his communication, however, is a case by Dr. Chambers, treated at the St. George's Hospital, where the inflammatory nature of the purpura was evident.

*Dr. Chambers' Case.* This was a girl, 16 years of age, who came into the hospital with spots of purpura on the legs, thighs and fore-arms. Pulse full and frequent—tongue clean, bowels open. She had been ill

---

* Med. and Phys Journal, November, 1826.

nine months with repeated crops of hæmorrhagic spots. Venesect. ad ℥x. To take infusion of roses and sulphate of magnesia thrice a day—and to apply a spirituous lotion to the parts. The blood was slightly inflamed. The spots began to fade in two days, and in a week afterwards returned, with blood in the urine and stools. She was then purged with calomel and sulphate of magnesia, and next day bled to eight ounces. After the bleeding, the pulse became small and very frequent, and the patient continued weak, some spots appearing and disappearing from time to time. In three weeks after entering the hospital, she complained of severe pain, tenderness, and tension across the umbilical region—the motions being green—pulse 110, and sharp—tongue furred—skin cool and pale. Bled to ten ounces—saline draughts, with Battley's liq. opii sed. 4tis horis. The blood was highly inflamed —pain relieved by the venesection, but still existing in the right iliac region. Repeated the venesection to ten ounces. Saline draughts, with digitalis and laudanum, were given, and she was put on a milk diet. The blood was not inflamed, but the pain and tenderness were relieved for a day, when they returned, and 16 leeches were applied. She was purged, and once more bled. In ten days from this time she was reported free from complaint. Spots of purpura, however, continued to appear from time to time, and were always removed by mercurial purgatives. On the 12th of May, or 48 days after coming into the hospital, she was attacked with violent pain in the occiput and back of the neck, accompanied with throbbing and some delirium, small quick pulse, dry and warm skin, furred tongue, &c. She was cupped, leeched, blistered, purged, had cold to the head, and antimony and digitalis internally, but in three days she died comatose.

On *Dissection*, some of the convolutions of the small intestines were found agglutinated by adhesive inflammation, but no other abdominal disease. It is not stated whether or not the mucous membrane was examined. It was in this part, of course, that disease, if any, was to be expected. In the thorax, they found an ounce of serum in the pericardium—the left ventricle of the heart dilated to nearly twice its natural size—the muscular parietes attenuated—in the corresponding auricle a morbid growth of a condylomatous nature. The mitral valve was much thicker than natural, but not ossified. In the head, the whole arachnoid membrane on the upper part of both hemispheres was covered with a lamina of coagulated lymph, the product of inflammation, and evidently the cause of death.

### 16. MEMOIR ON ABSCESSES OF THE LIVER. BY M. LOUIS.*

[La Charité.]

A question has often been started by several physicians, whether an abscess, containing homogeneous, unctuous—in one word, *laudable pus*, can take place in the parenchymatous structure of the liver, or only

* Repertoire, No. 2, 1826.

in the cellular membrane, between its peritoneal covering and glandular texture? The answer in the affirmative will be given by most practitioners who have resided long between the tropics, especially in the eastern world. But assuredly the greater number of abscesses which we find there, do not contain good pus, but a fluid more resembling the washings of beef. M. Louis observes, that the history of hepatitis is yet very defective, whether we look to the causes, the symptoms, or the terminations. In 430 dissections, where every organ of the body was accurately examined, M. Louis found five examples of purulent abscess in the *substance* of the liver, and not one in the coverings. Of these we shall give a succinct account.

*Case* 1. A young woman, 21 years of age, of delicate constitution, but not at all emaciated, was admitted into La Charité on the 19th of December, 1822. She had gone through the process of parturition 24 days previously, the labour being natural and easy, and the patient having left the St. Louis, where she was confined, seven days after her accouchement. She caught cold on her way home, and this was the cause of her present illness, which commenced with acute pain in the præcordial region, followed, in the evening, by a succession of shiverings, fever, and perspiration. The pain ceased on the following day, and four days afterwards she was seized with acute pain in the right side of the chest, which was much augmented by the slightest cough. The shiverings had returned each evening, the thirst was considerable, the appetite lost, and there was some nausea when she took drink. She had also a diarrhœa, but without any pain in the bowels. In a few hours after entering the hospital, a great number of leeches had been applied to the affected side, and M. Louis found her the next day, (20th), presenting the following symptoms:—character of distress in the countenance—slight tinge of yellow on the skin—great sense of debility—lumbar pains—intellectual functions undisturbed—no head-ache—tongue soft, red and dry in the middle, white and moist on the sides—anorexia—thirst—pains in the epigastrium, augmented by cold drink—lies on the *left* side—breathing short, 45 in the minute—pain under the right mamma, deep-seated, and increased by pressure—but little respiratory noise in this part of the chest, no egophony—heat elevated—pulse quick, 120 in the minute, but not sharp—great sensibility to cold air—continuance of the diarrhœa—had had emulsions and lavements in the night.

21st. The nose, lips, and cheeks of a blue cast, the rest of the face pale—debility extreme—tongue dry—pulse extremely feeble and small. The cough was troublesome through the day—much restlessness in the night, talking incessantly about her complaint. She died the next day, (23rd), after being some hours delirious.

*Dissection.* There was nothing worthy of notice in the head. In the chest, the heart and aorta were sound. In the left lung there was a crude tubercle at the upper part, and some grey granulations throughout the lung generally. In the right side, the pleura pulmonalis, costalis, and diaphragmatica, were covered by a soft, yellow, false membrane, with five or six ounces of serous fluid effused in this cavity. No engorgement of this lung, but the bronchiæ were of a brownish red colour throughout. In the abdomen, the stomach and intestines were much distended—the form and size of the liver were natural—its middle lobe of an orange colour—the great lobe was intensely red throughout its whole substance, and softer than natural. In the centre of this lobe was found a cavity, capable of containing an ordinary hen's egg, filled with pus of a slightly greenish hue, of a good consistence, and without any smell. The cavity was lined with a false membrane, of a greyish white colour, being about the thickness of the mucous membrane of the stomach. In the vicinity of this abscess the liver presented the same appearance as in other parts of its structure, except that it was somewhat softer, for the space of three or four lines, round the parietes of the abscess, than in the rest of the organ. There was nothing remarkable in the biliary ducts, and the gall-bladder contained some bile as thick as molasses. The mucous membrane of the stomach was softened in some places, and indurated in others, especially near the pylorus. There was evidence of inflammation in some of the small intestines. There was no other morbid appearance of any consequence in the body, except some traces of inflammation in the uterus.

*Remarks.* That this was an abscess in the substance of the liver there can be no reason to doubt; but that it was not of so recent a formation as the inflammation in the rest of the liver, there is every reason to believe, from the state of the parietes of the abscess. The death of the patient we cannot attribute to the hepatic inflammation or suppuration; nor yet, perhaps, to the pulmonic, but chiefly to the gastric, in combination with the other phlogoses, so strongly developed in this patient. The severe pain in the epigastrium, the nausea, and the diarrhœa, were symptoms of the gastritis and enteritis. However, there was quite enough to account for the death of the patient, on the whole.

*Case* 2. A man, 23 years of age, of apparently good constitution, well-made, active, and sober in his habits, experienced, in March, 1824, all the symptoms of an inflammation of the mucous membrane of the stomach and bowels. The complaint soon gave way to energetic antiphlogistic treatment, and he left the hospital in a month, with only a slight diarrhœa. This last continued for two years, without any disturbance of the digestive functions, or prevention of his usual avocations. When he returned, on the 24th of March, 1826, he had lost none of his embonpoint. He reported, that during the last two years he had had frequent head-aches, with giddiness, for which leeches were often applied. During the last two months, the head affections were constant, and for ten days previously, they were accompanied by other severe symptoms, which confined him to his bed. On the 22d of February, when the present illness commenced, he had shiverings, followed by thirst, anorexia, &c. The shiverings did not return, but a continued fever-heat succeeded. During the last five or six days, there were nausea, slight pains in the bowels, constipation, restless nights, some cough. He had been twice bled, without relief.

25th *March*. Expression of suffering in the countenance—intellectual functions not free, the least attempt to exert the memory being accompanied by distress. There is some cephalalgia—giddiness on sitting up—an ulcer at the bend of the left arm, where he was bled—and an abscess on the spot where the vein was opened in the right arm—anorexia—thirst—pasty mouth—tongue dry in the centre, white and moist at the sides—pain in the epigastrium on pressure—three liquid motions in the last 24 hours—pulse full, and very frequent (144 in the minute)—heat not very great—no cough at present—universal malaise, without the power of describing what it was. Bled from the foot—lowest scale of diet—diluents.

He had six stools in the night, which was spent in great agitation. Next morning (26th), the cheeks are red—the whole surface of the body tinged yellow—thirst more urgent—epigastrium very little painful, the supra-pubic region more so—the right hypochondrium tense, and very tender on pressure—pulse 116—debility increased. Twelve leeches to the anus—ptisans. 27th. Much blood flowed from the leech-bites—the yellow colour of the skin is deeper—the patient only complains of pain when the abdomen is pressed—the cheeks are of a purple-red colour—the physiognomy sombre—pulse 108—skin dry and hot. In the course of this day the stools became involuntary. 28th. The yellow colour still more intense—constant somnolency. Bled to ten ounces. The blood was covered with a yellow buff—stools involuntary. 29th. Was incapable of speech. The tension in the hypochondrium was increased, and the whole abdomen very sensible on pressure. The respiratory noise was very loud in the left side of the chest; very dull in the right. The same with percussion. Death closed the scene before daylight of the 30th.

*Dissection*. There were appearances of inflammation in the brain and its membranes. There was also inflammation, as well as infiltration, in the throat. The heart was sound. In the left lung, near its summit, were two broken-down tubercles, containing fluid purulent matters. In the right lung were also two excavations. The right lung was covered everywhere by a false membrane, which adhered slightly, except at one place, to all the neighbouring parts. In the bag formed by the pulmonary and costal false membrane, half a pint of thick purulent matter was contained.

The Liver was voluminous, and descended four inches below the margin of the ribs; it was very red, and much softer than natural. Between the liver and diaphragm a quantity of pus was seen, which issued from a large abscess in the substance of the organ. The cavity of the abscess was divided into two by an incomplete partition: one was as large as a goose's egg—the other would have contained a hen's egg. These cavities contained, along with some good pus, a quantity of troubled fluid, of a greenish colour, and resembling curds and whey. The abscess was lined by a false membrane of no great consistence, and easily separable from the substance of the liver, with which it was in contact. The bile in the gall-bladder was more viscid than natural—nothing particular in the biliary ducts. There were marks of inflammation in the mucous membrane of the stomach. The kidneys were double their natural size.

M. Louis has made a great many comments on this case, but none of them of any particular interest: we shall therefore proceed to facts.

*Case* 3. A medical student, 22 years of age, of rather weakly constitution, was seized, shortly after his arrival in Paris, in November, 1821, with diarrhœa, which continued six months, and did not entirely vanish till after his return to the country. In January, 1823, the bowel-complaint was renewed, and again persisted pretty severely for six weeks, when it ceased. The diarrhœa disappearing, he complained of pain in the right hypochondrium, which became suddenly aggravated, with a slight icteritious tinge of the skin. M. Chomel was now summoned to the patient, and found him in the above condition—his skin and urine being yellow—the hypochondriac pain very violent. In the course of the next day he passed a quantity of black coagulated blood by stool. This discharge continued three days, and the quantity lost was estimated at nine or ten pounds. Cold lavements, ices internally, and opium were prescribed. On the 7th of April, M. Louis saw the patient for the first time. Debility and emaciation were great—the sclerotic coats of the eyes were of a greenish hue, all the rest of the body of a pale yellow colour—tongue pale and moist—little thirst—complete anorexia—belly drawn in—right hypochondrium tender, but not much so—fæcal matters of a yellowish green colour, puriform, and of a heavy odour. He had four or five stools daily since the cessation of the hæmorrhage—respiration natural, pulse much accelerated, small, and feeble. During the succeeding days, the pains in the hypochondrium diminished—and on the 12th were much exasperated, and were alleviated by another large discharge of blood from the intestines. This sanguineous discharge ceasing, the stools again became puriform. Towards the beginning of May, the patient's appetite had returned, and he ate greedily, without any weight in the epigastrium, diarrhœa, or pain any where. The skin was now but slightly tinted yellow, and the pulse had come down to its natural level. The debility was diminishing—he was able to walk a little in his chamber, and he got sleep by means of a gentle opiate at night. He talked of returning to his friends in the country, but not being equal to that enterprize, and all his finances exhausted, he entered the hôpital La Charité, under M. Chomel. He did not feel much fatigued by the removal, and only complained of debility when placed in a warm bed in the ward St. Jean. But his emaciation was extreme—tongue pale and moist—belly torpid—pulse calm. On the third day after entering the hospital, he took some chicken with a relish, but he soon fell off, became oppressed in his breathing, and died on the 9th of May, while M. Louis was going round the ward.

*Dissection.* Passing over the appearances in the brain, which was a little softer than natural, we come to the right lung, which was hepatized, and covered by false membranes of soft consistence. The liver did not descend below its usual depth in the abdomen—was of its natural size, but of a deep red colour, interspersed with yellow spots. On making deep incisions in various directions, there were seen a number of roundish yellow patches, from three to eight lines in diameter, and occupying the greater part of the parenchymatous substance. These patches were found to be an assemblage of globular cysts, from two to three lines in diameter, containing matter of a greenish yellow colour, thick, and decidedly purulent. In some places, the pus was broken from the cysts, and extravasated into irregular excavations, five or six lines in diameter. On making a deep incision, an excavation, capable of containing a walnut, was found, filled with black coagulated blood, disposed in concentric layers. This cavity was lined by a double false membrane, the inner fold of which was red, while the outer was of a greyish white, and strongly adherent to the parenchymatous structure of the liver. There was no direct communication between this cavity and the vena portæ, or biliary ducts, in which there was nothing remarkable to be seen. The mucous membrane of the stomach was of greyish colour throughout, but of normal thickness and consistence. No appearance of inflammation in the duodenum or small intestines, except near the ileo-cæcal valve, were there was evidence of six ulcerations perfectly cicatrized, of oval forms, from six to eight lines in diameter. These cicatrices were at once distinguishable by depressions, evident to the touch as well as to the eye. The mucous membrane of the large intestine was very much softened, and the descending colon, sigmoid flexure, and rectum, were full of small ulcerations, some of them penetrating to the muscular coat. The other viscera were of a natural appearance.

This is an interesting case. There can be little doubt that the black blood discharged in such large quantities, *per anum*, proceeded from the liver. The pathological condition of the small and large intestines is also worthy of notice. But on these points we leave our readers to their own reflections.

*Case* 4. A sailor, aged 50 years, lame, and paralyzed of one leg from youth, was received into La Charité, on the 15th of October, 1825. He had been ailing for three or four years, but had been much worse for the preceding seventeen days. He attributed his illness to moral causes—anxiety of mind, and profound grief. The digestive organs had been much deranged during the four years preceding. He had had slight pains in the left hypochondrium—seldom nausea or diarrhœa, and alternately he gained and lost flesh. At the commencement of his present illness, he experienced febrile heat, without chills, yellow suffusion over the whole body, complete anorexia, sharp pains in the epigastrium and left hypochondrium, slight oppression. These symptoms had persisted up till the moment of his reception into the hospital, with the addition of diarrhœa and some nausea during the last eight days. There was now deep jaundice of the skin and eyes—cephalalgia—pains in the limbs and loins—tongue coated—complete anorexia—ardent thirst—gravitative pain in the epigastrium—apparent enlargement in the region of the liver, below the false ribs, extending across the epigastrium to the left side—accelerated breathing—respiratory noise but faintly heard by the stethoscope—pulse 120 in the minute, strong and hard—skin dry and hot—bowels not opened for 36 hours. *Venesection to ten ounces—diluents—low diet.* From this time till the 30th, the day of his death, the following symptoms were noticed, *viz.* continuation of the jaundice—severe pain, on the 17th, in the region of the gall-bladder, which continued more or less afterwards —no nausea or vomiting—stools only procured by glysters—urine abundant, but of very deep colour—crepitation in the left side of the chest, and afterwards in the right. On the 28th, delirium commenced, and on the 30th death took place.

*Dissection.* Some traces of inflammatory action in the brain and its membranes. Lungs adherent—the upper portions crepitous, but the inferior gorged with blood, and easily torn. The air-cells were much dilated—the bronchia red, thickened, and enlarged, containing some mucus—heart adherent to the pericardium by cellular shreds. In the abdomen, the liver was found to be rather larger than natural, and presented several yellow patches on its surface. In its substance were a number of small encysted abscesses, from four to five lines in diameter, containing thick pus, of a greenish yellow colour, and lined with a regular white membrane, which was soft, and easily separated from the surrounding parenchymatous structure of the liver. The substance of this organ was everywhere softened, and redder than natural. The gall-bladder was very small, contained only some mucus, and its duct obliterated. Its parietes were more than a line in thickness. In the cystic duct was a calculus, eight lines in diameter, compressing the hepatic duct. In the stomach there were some places inflamed and softened—some perfectly sound—and others completely ulcerated. There was some evidence of inflammation in the mucous membrane of the small intestines, and unequivocal proof of the same in the colon. The other viscera natural, except the spleen, which was twice its natural size.

The above case offers materials for some useful reflections. There can be little doubt that the original cause of this series of organic changes may be traced to the mental anxiety, of which this unhappy man was the prey. This state of mind led to the disorder of the digestive organs, and ultimately to the structural disease in the same organs, as well as in the heart. The changes found after death accord with the symptoms during life, with the exception of the *apparent enlargement of the liver* felt below the margin of the ribs, and extending across the epigastrium. We have known many instances where deception of this kind was occasioned by a loaded transverse arch of the colon, giving the feel of an enlarged liver. Even the upper belly of the rectus muscle, on the right side, will deceive the incautious examiner, by its hardness and prominence, when the fingers are thrust hard under the ribs.

*Case* 5. A gardener, 55 years of age, of middling constitution, and not addicted to excesses in wine, but rather short of breath for many years, was subject to annual attacks of pulmonary catarrh for the last six years. The present attack had lasted five months, when he entered La Charité, 28th March, 1823. To this was added, recently, a pain in the left side of the chest—chills and heats alternately—restlessness and wandering in the night—anorexia and thirst. Emaciation had lately made progress. 29th. Cephalalgia—pain in the limbs and loins—great sense of debility—intense thirst—mouth pasty—tongue white and coated, except at the point and sides—constipation of bowels—pain in the left side, increased by coughing; crepitation of the same side—percussion obscure—respiration tracheal. Nothing remarkable on the right side—expectoration mucous, with some globules of semi-transparent matter—pulse 80. *Ptisans—a blister—venesection to ten ounces.* The dyspnœa was relieved, and the pain quickly disappeared. The patient sometimes had a little appetite, but generally there was complete anorexia. On the 11th April, some diarrhœa came on, and continued more or less till his death. Towards the latter end of May, jaundice, of rather a deep hue, supervened, and continued to increase in intensity, till the end—at the same time, he complained of pains in the right side of the epigastrium. On the 25th May, these pains were very acute, and pressure was insupportable. By the 1st of June, these pains occupied a larger extent, occupying the whole of the right hypochondrium, and part of the epigastrium, also a part of the right side of the chest. These continued without interruption till his death, that is, for the space of a month. The pulse came down to 60 in the minute, and did not latterly exceed 65. Debility made rapid progress while in the hospital, so that at last he could hardly move his arm. He lay on the right side, with his head low, and made but little complaint. The intellectual functions were little disturbed till within a few days of his end, when delirium came on. We need not allude to the medical treatment, since nothing could be done but mitigating symptoms.

*Dissection*, 1*st July*. The left lung adhered to the ribs, and its upper portion was covered with a semi-cartilaginous false membrane, under which the parenchymatous structure of the lungs was firm, and of a dark grey colour. In the right lung there was a good deal of infiltration of a reddish fluid, and eight or ten ounces of effusion into that side of the chest. The bronchiæ were red on the left side. The liver did not descend below the edges of the ribs. It was of a variegated colour, and contained from 30 to 40 small cavities, varying in size, from that of a pea to that of a walnut, filled with greenish pus, of moderate consistence. These small abscesses were not encysted, their parietes being merely the parenchymatous substance of the lungs a little consolidated and reticulated. The structure of the liver generally was softened. There were some calculi in the gall-bladder, and its mucous membrane was thickened and ulcerated. There was nothing worthy of notice in the other viscera.

M. Louis makes some observations on the symptoms of hepatitis, in which we agree with him; and some in which we differ. He properly observes, in the first place, that in none of the cases above detailed was the disease of the liver uncomplicated with affections of other organs. As to the symptoms, however, which unequivocally appertain to the hepatitis, it may be remarked that, in four cases out of the five, there was jaundice, with pain in the right hypochondrium—and in two only was there *tension* in this part. An union of these three symptoms M. Louis observes, characterises hepatitis, with sufficient accuracy, but no one, nor even two of them, are satisfactory proofs that the liver is inflamed. Jaundice may obtain without hepatitis: and as for *pain*, it may be seated in neighbouring parts, though apparently in the liver. "But," says he, "it is quite otherwise when pain and jaundice supervene in the course of chronic maladies—for then it is a proof of inflammation of the liver." Of this we entertain doubt:—for the pain may still be in other parts than the liver, and the jaundice may be occasioned by mechanical obstruction of the ducts, from the pressure of a tubercle or other tumour, quite unconnected with any decided inflammation.

None of M. Louis' patients experienced pain in the right shoulder—"and we are very doubtful," says he, "whether this symptom, so much insisted on by authors, be really indicative of hepatitis. Perhaps, in those cases where it is present, it is attributable to some affection of the lung or pleura of that side." Perhaps it is; but, at all events, it is far from being so common, either in acute or chronic hepatitis, as is generally believed.

In respect to the *causes* of hepatitis, our author disbelieves that it is more frequent in hot than in cold climates—and this on the authority of P. Frank, who, it seems, has practised in climates the most opposite. It is quite unnecessary to contradict M. Frank in this place—since English physicians and surgeons know better. It is true this disease is not equally prevalent in all hot climates—but take India, for example, and we shall find that hepatic affection is just as characteristic of that country as pulmonic affection is of England.

"It has been insisted on of late," says M. Louis, "that, where hepatitis is not directly caused by external violence, it is always owing to inflammation of the mucous membrane of the duodenum." This doctrine, we believe, is that of the younger Broussais. It is scarcely worth refuting. M. Louis dryly observes, that, in four cases out of the five here detailed, the mucous membrane of the duodenum was only inflamed in one.

Blows on the head have been enumerated among the causes which produce abscess in the liver. Our author does not believe in the truth of this point of etiology; but certainly observation (and nothing but this could ever have led to the belief) has shewn that, in blows and injuries of the head, there is more frequently abscess of the liver induced than of any other organ. Because we cannot account for such a circumstance, are we to deny it in the face of facts?

In respect to discoloration and softening of the parenchymatous structure of the liver, our author wisely acknowledges that, in the present state of our knowledge, we cannot set them down as proofs of preceding inflammation.

One remark of M. Louis' is very curious, namely, that abscesses in the liver are incurable, since he has never been able to find a cicatrix in any dead body. But those who have practised in our Indian colonies know that abscesses of the liver will heal occasionally, whether they are opened externally, or make their way into the intestines. The most unequivocal proofs of this are every day seen in the East Indies. We do not, indeed, maintain that abscess, the result of chronic or sub-acute inflammation, in this or in any other country, where there is an encysted character, as in all the above cases, and where there is general tendency to organic disease, is likely to heal. But where acute inflammation terminates, in the course of fourteen or fifteen days, in suppuration, there we have a chance of cure, especially if the tumour points externally, and the knife is applied in proper time. Fortunate results are frequently seen under such circumstances.

Upon the whole, we feel indebted to M. Louis for his very minute and circumstantial detail of the symptoms and dissections in the above cases, and have pleasure in giving them a registry in our Journal.

### 17. MR. STONE ON POLYPUS UTERI.*

The object of Mr. Stone, in this paper, is to prove that, after the removal of firm polypous tumours, *attached by a narrow neck* to the uterus, although the greater portion, or the whole of the peduncle by which they were connected should remain above the ligature, yet that this will be thrown off by nature, and the organ left in a healthy state. This, however, will not be the case where the tumour is of softer consistence, and where the attachment to the uterus is nearly of the same diameter with the tumour itself. In these last cases, it appears that a great part of the uterus is involved in disease, and the tumour is soon reproduced. Examples of this reproduction are given by our author. But although the tumour, under such circumstances, is almost sure to grow again, Mr. Stone thinks the removal of it is proper, in order to prolong life where we are unable to effect a complete cure.

*Case* 1. Mrs. B. aged 47 years, had been suffering from a coloured discharge for two years, attended by occasional coagula of blood. Menstruation had ceased. On examination, a polypus, the size of a plover's egg, was found in the vagina, its neck being surrounded by

* Mr. Stone on separation of the neck of polypus of the uterus, after the removal of the body of the tumour. Med. and Phys. Journal, No. 5, New Series.

the os uteri. A ligature was passed round the tumour, and tied by means of a canula. On the two succeeding days it was tightened, and, on the third, it came away, the ligature still remaining upon the neck of the tumour, and there being a portion, half an inch in extent, above the point of the peduncle round which the ligature had been applied. In a short time the discharge ceased, and the woman recovered. This happened in 1819, at the St. George's and St. James's Dispensary.

*Case* 2. A poor unmarried woman came to town for advice in 1817, She had laboured many years under profuse discharge from the vagina. Mr. Clark discovered that she had a polypus as big as a child's head. The polypus was removed; but the woman was attacked with symptoms of peritoneal inflammation, and died. On examination, the os uteri was found somewhat more dilated than usual, but otherwise in a natural state. The neck of the tumour, which was of a very small size, compared with the tumour itself, was still attached to the fundus uteri, but the point of attachment was very small, being much thinner than any other part of the neck of the tumour, and nearly separated from the uterus. The preparation is in the author's collection.

---

### 18. TETANUS.*

Any contribution to the pathology of a disease so involved in obscurity and so indomitable as tetanus, must be both of interest and importance. Mr. Swan of Lincoln, as our readers will recollect, has brought forward cases to prove that the cause of tetanus consists in an irritation and inflammation of the ganglia—others are of opinion that the spinal marrow is the seat of the affection. The following cases which occurred in public institutions go to favour the latter hypothesis, but not to *overthrow* the former, as we shall see presently.

*Case* 1. Coquillard, a hair-dresser, of bilious temperament but strong constitution, was admitted into the Saint-Louis under M. M. Cloquet and Richerand, on the 8th May, 1826. On examination there was found a carbuncle on the upper and inner part of the left leg. M. Richerand made a crucial incision into the tumour, and poultices with the warm bath were ordered. All went on well till the 15th, when trismus made its appearance. On the 19th tetanic paroxysms developed themselves;—*acetat. morphin.* gr. ij.—*venesection.* 20th. Opisthotonos—*acetate of lead—acetate of morphine to the wound.* 21st. M. Cloquet made large and deep incisions on each side of the spinal column from the occiput to the loins—cupping glasses were then employed. 23d. The patient sunk during a convulsion.

*Dissection, 40 hours after death.* The veins of the spinal dura mater were much congested—the dura mater itself was injected, so was the

---

* Messrs. Monot and Jobert. Hôp. St. Louis et St. Antoine. N. Bibliothéque, Août, 1826.

pia mater—the membrana propria of the marrow was red and thickened On cutting into the marrow, it was found red and so soft, except in the centre, as to be almost diffluent—this state obtained from the fourth cervical to the fourth or fifth dorsal vertebra. The dura and pia mater of the brain were much injected. The branch of the sciatic nerve distributed to the site of the carbuncle was encircled by quite a vascular net-work—mucous membrane of the stomach and bowels inflamed and even nearly ulcerated in parts.

*Case* 2. Pradot, æt. 22, a mason, entered the Saint-Antoine, August 18th, 1822, for an extensive injury of the arm. On the 22d, four days after admission, tetanus shewed itself; the symptoms were similar to those in the last case. He took two doses of 50 drops of laudanum. On the 29th he died.

*Dissection, 24 hours after death.* The membranes of the medulla, with the exception of the dura mater, were injected; so was the medulla itself—nodus cerebri red and harder than natural—all the nerves were reddened.

*Remarks.* It will be observed, that in both these cases there is no mention made of the state of the ganglionic system. In the first case it seems almost probable that this system was primarily affected, for the surface of the stomach and bowels with which it is so closely connected, was much disorganized. The dissection in the second case appears to have been so superficial that little or no inference can be drawn from it. It is rather curious that in one instance there should be morbid *softening* of the medulla, and, in the other, morbid *hardening* of the nodus cerebri. There was evident inflammation of the membranes and of the substance of the medulla in both, the indication, therefore, both from the results of these, and indeed from those of the majority of cases of tetanus, would be active antiphlogistic treatment. Opium, however, and that in larger doses than are usually employed in this country, must not be neglected.

It is also to be remembered that, from the intimate sympathy existing between the ganglionic and spinal nerves, any irritation in the former may very readily excite morbid phenomena in the latter. If worms in the primæ viæ produce convulsions, so irritation of the semilunar ganglia may produce tetanus. In either case, there may be nothing found on dissection, as many post mortem examinations prove. In other instances, the irritation may go on to inflammation, and then the traces of this state may be found in one or both of the systems of nerves.

N. B. We have been obliged to transfer some articles which ought to have come into this department of the Journal to the succeeding division of the Periscope, in consequence of their late arrival. This, however, is not attended with any inconvenience, as they will still be distinguished as Hospital Reports.

